# Circulating vitamin levels mediate the causal relationship between gut microbiota and cholecystitis: a two-step bidirectional Mendelian randomization study

**DOI:** 10.3389/fnut.2023.1268893

**Published:** 2023-09-26

**Authors:** Changhong Miao, Lu Xiao, Xinyi Xu, Shuoxuan Huang, Jiajin Liu, Kuang Chen

**Affiliations:** ^1^First Teaching Hospital of Tianjin University of Traditional Chinese Medicine, Tianjin, China; ^2^National Clinical Research Center for Chinese Medicine Acupuncture and Moxibustion, Tianjin, China

**Keywords:** gut microbiota, circulating vitamin levels, cholecystitis, Mendelian randomization, causal relationship

## Abstract

**Background:**

The relationship between gut microbiota and the occurrence of cholecystitis remains unclear. Existing research lacks a clear understanding of how circulating vitamin levels modulate this relationship. Therefore, our study aims to investigate whether circulating vitamin levels mediate the causal relationship between gut microbiota and cholecystitis using a two-step bidirectional Mendelian randomization approach.

**Methods:**

In this study, we initially employed Linkage Disequilibrium Score Regression (LDSC) analysis to assess the genetic correlation of five circulating vitamin level genome-wide association study (GWAS) summary datasets, thereby avoiding potential sample overlap. Subsequently, we conducted a two-step analysis to investigate the causal effects between gut microbiota and cholecystitis. In the second step, we explored the causal relationship between circulating vitamin levels and cholecystitis and identified the mediating role of vitamin D. The primary method used for causal analysis was the inverse variance-weighted approach. We performed additional sensitivity analyses to ensure result robustness, including the cML-MA method and reverse Mendelian randomization (MR) analysis.

**Results:**

An increment of one standard deviation in *RuminococcaceaeUCG003* was associated with a 25% increased risk of cholecystitis (OR = 1.25, 95%CI = 1.01–1.54, *p* = 0.04), along with a 3% decrease in 25-hydroxyvitamin D levels (OR = 0.97, 95%CI = 0.944–0.998, *p* = 0.04). However, following the rigorous Bonferroni correction, every one standard deviation decrease in circulating vitamin D levels was associated with a 33% increased risk of cholecystitis (OR = 0.67, 95%CI = 0.49–0.90, *p* = 0.008, P_adjust_ = 0.04). Thus, the potential link between gut microbiota and cholecystitis risk might be mediated by circulating vitamin D levels (proportion mediated = 5.5%). Sensitivity analyses provided no evidence of pleiotropy.

**Conclusion:**

Our study results suggest that an elevated abundance of specific gut microbiota is associated with an increased susceptibility to cholecystitis, with the causal relationship being mediated by circulating vitamin D levels. Further large-scale randomized controlled trials are necessary to validate the causal effects of gut microbiota on cholecystitis risk. This study provides novel insights into cholecystitis prevention through the regulation of gut microbiota.

## 1. Introduction

Cholecystitis is primarily an acute or chronic inflammatory reaction in the wall of the gallbladder, usually caused by gallstones or a bacterial infection of the gallbladder duct (1). Epidemiological data indicates that approximately 20 million people in the United States are affected by gallstones (2), with 1 to 4% of gallstone patients developing conditions such as chronic cholecystitis, acute cholecystitis, or gallstone-induced pancreatitis (3). Furthermore, delayed treatment of biliary infections can still lead to systemic inflammatory reactions and potentially fatal outcomes, with mortality rates ranging from 11 to 27% (4). Modern comprehensive studies on cholecystitis have revealed a significant association between its etiology and factors such as diet and lifestyle. Therefore, investigating early intervention and preventive strategies for cholecystitis remains crucial in mitigating the societal health burden (5).

Bile is a biofluid synthesized in the liver, stored, and concentrated in the gallbladder, where it is released into the intestine, specifically the duodenum, following meals. Alterations in the gallbladder due to disease can lead to changes in the gut microbiota composition as a result of bile influence on the gut ecosystem (6). Molinero et al. conducted 16S rRNA sequencing (16S rRNA sequencing is a method to study and identify bacteria and archaea by analyzing a specific genetic marker called the 16S ribosomal RNA gene. It helps reveal the composition and diversity of gut microbiota in various environments) and metagenomic sequencing (Metagenomic sequencing is a method to study all genetic material in gut microbiota, offering insights into diversity and functional potential) of bile samples from the gallbladder. Their findings revealed similarities in the distribution of major functional categories in the biliary metagenome to those described in the human gut microbiota, suggesting potential associations between the two (7). The human gut microbiota is composed of trillions of microorganisms. These microorganisms in the gut can undergo translocation through the duodenum via the Oddi sphincter or enter the liver through the bloodstream, subsequently being excreted into the bile. This process facilitates bacterial migration and colonization within the bile system (8). Recent research has indicated a significant association between the gut microbiota and the occurrence of acute cholecystitis. Likewise, Liu et al. utilized 16S rRNA and metagenomic sequencing techniques, obtaining a total of 185,713 high-quality sequence reads from fecal samples collected from 15 patients and 13 healthy controls. Their findings revealed a correlation between the gut microbial *family Enterobacteriaceae* and acute cholecystitis (9). Furthermore, several studies have proposed a significant association between *Helicobacter pylori* infection and cholecystitis. The underlying mechanism may involve *Helicobacter pylori*’s capability to infect the biliary system, leading to chronic inflammation of its mucosa. Consequently, this can impair bile acid secretion and reduce calcium salt solubility, thereby increasing the likelihood of gallstone formation (10–12).

It is well known that the human immune system relies on various micronutrients, such as vitamins A, D, C, E, B6, B12, and folate, as well as zinc, iron, copper, and selenium. These micronutrients play critical and synergistic roles in various stages of the immune response, ensuring the normal functioning of physical barriers and immune cells (13). Given this, there may exist potential regulatory mechanisms between circulating vitamin levels in the bloodstream and the occurrence of cholecystitis.

Existing research has also substantially demonstrated the interaction between the gut microbiota and circulating vitamin levels. The gut microbiota has the capacity to produce, consume, or compete for vitamin B, and supplementation or deficiency of vitamin B may impact the growth of specific bacteria, leading to changes in the composition of the gut microbiota (14). Li and Didier, for instance, found that antioxidant vitamins A, E, and β-carotene can improve gut barrier function and maintain normal immune system function by modulating the composition and metabolic activity of the gut microbiota, or by directly influencing the body’s immune response in response to oxidative stress (15, 16). Luthold and colleagues, in a study investigating vitamin D intake in 150 healthy adults, demonstrated that the role of vitamin D in maintaining immune homeostasis partly involves interactions with the gut microbiota (17). Interestingly, an observational study indicated that circulating vitamin C levels have a protective effect against cholesterol gallstones (18). Notably, vitamin D exerts potent regulatory effects on both innate and adaptive immunity (19), with research suggesting that maintaining serum 25(OH)D concentrations above 40 ng/mL can significantly reduce symptomatic diseases and decrease the prevalence of infections and chronic conditions (20).

Therefore, it is crucial to determine whether circulating vitamin levels (vitamins A, B12, C, D, and E) in the bloodstream interact with the gut microbiota in the development of cholecystitis. Unfortunately, these studies still face the risk of confounding factors and reverse causality, making the potential causal relationship between the gut microbiota, circulating vitamin levels, and cholecystitis unclear. Previously, a Mendelian randomization (MR) study involving Genome-wide association study (GWAS) of the gut microbiota from 18,340 individuals and GWAS data of vitamin D from 417,580 individuals showed a causal relationship between *Erysipelotrichales* and circulating vitamin D levels (The effect size of the main MR analysis method result:β = −0.06, The *p*-value after multiple correction using the False Discovery Rate (FDR) method: P_FDR_ = 1.93 × 10^−4^) (21). However, to ensure the accuracy of the results, this study will conduct further research using a larger-scale GWAS meta-analysis of circulating vitamin D levels.

MR is a robust methodological framework that employs genetic variation as instrumental variables. By conducting a comprehensive analysis that integrates data from GWAS and gut microbiota, we can identify genetic variants closely associated with gut microbiota traits. This approach allows us to overcome limitations inherent in traditional observational studies, including confounding factors and reverse causality, thus providing more reliable evidence for causal inference (22). Furthermore, this study employs a series of sensitivity analyses to ensure the stability and reliability of the results. It also employs the cML-MA method to eliminate potential pleiotropy, both coherent and incoherent. The primary objective of this research is to assess the causal impact of gut microbiota on cholecystitis and the mediating role of circulating vitamins. This study aims to advance our understanding of the mechanisms underlying cholecystitis and holds significance for the development of preventive and therapeutic interventions. Future research should involve large-scale clinical randomized controlled trials to validate the causal relationships between gut microbiota, circulating vitamin levels, and the risk of cholecystitis.

## 2. Materials and methods

### 2.1. Study design

This study employs a two-sample two-step MR design based on GWAS summary data. The study follows the latest guidelines for MR analysis, as outlined in the STROBE-MR guidelines, and operates under three fundamental assumptions: 1. the instrumental variables are closely associated with gut microbiota; 2. the selected instrumental variables are independent of any potential confounding factors; and 3. the genetic variations are unrelated to cholecystitis, except through their association with gut microbiota. Additionally, the absence of statistical interaction is required to meet other assumptions (23).

The two-step MR analysis is conducted in this study. In the first step, MR analysis between gut microbiota and cholecystitis is performed to obtain the effect size β_1_. In the second step, MR analysis is conducted separately for different circulating vitamin levels (vitamin A, vitamin B_12_, vitamin C, vitamin D, and vitamin E) and their associations with cholecystitis. If multiple vitamins show associations with cholecystitis, the Bayesian model averaging method is employed for comprehensive analysis. If only one circulating vitamin level is associated with cholecystitis, the effect size β_3_ is obtained after multiple corrections for the *p*-value. Subsequently, we perform MR analysis for gut microbiota and the mediator to determine the effect size β_2_.

**Figure 1:**
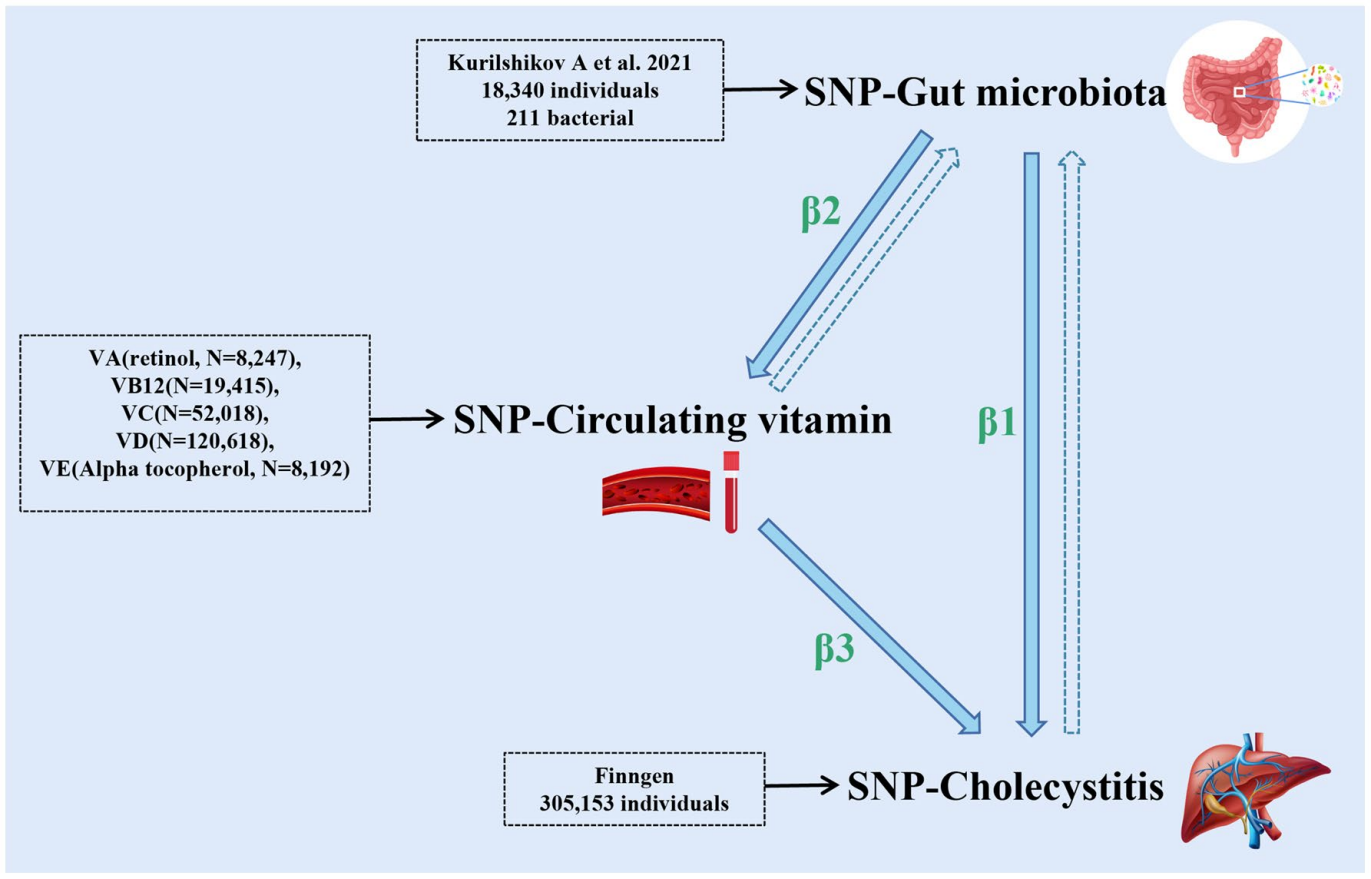
The schematic diagram of the study design (the figure illustrates the design and principles of this study, demonstrating the interactions among gut microbiota, cholecystitis, and multiple circulating vitamin levels). The dashed arrows in the figure represent the reverse Mendelian randomization studies conducted in this research.

The data used in this study are GWAS summary-level data, and thus all informed consents and ethical approvals have been previously obtained in the original studies. The schematic diagram of the study design is illustrated in Figure 1.

### 2.2. Data sources

The exposure data for this study were obtained from the largest-scale GWAS meta-analysis to date, comprising 24 cohorts. This study utilized 16S rRNA technology to analyze data closely associated with genetic variation for 211 gut microbial taxa, involving a total of 18,340 individuals (24). The outcome variable of interest, cholecystitis, was derived from GWAS summary data of the latest R8 version released by the FinnGen consortium, which included 3,770 cases and 301,383 controls. As for the intermediate variables, the study incorporated GWAS summary data for circulating vitamin A (retinol) levels from a sample of 8,247 individuals (25), GWAS summary data for circulating vitamin B12 levels from a sample of 19,415 individuals (26), and the most recent GWAS meta-analysis involving 52,018 individuals from five cohorts for circulating vitamin C levels (27). Additionally, the study utilized the latest meta-analysis of MR involving 120,618 individuals from eight cohorts for circulating vitamin D levels (25-hydroxyvitamin D) (28). Lastly, GWAS summary data for circulating vitamin E (α-tocopherol) levels from a sample of 8,192 individuals were also included. Further details regarding the specific sources of data for this study are presented in Table 1.

**Table 1:** This study presents a comprehensive summary of all data sources and information utilized.

Variable	GWAS ID/PMID	Data sources	Race	*N*	Web resource
Exposure					
Gut microbiota	PMID: 33462485	MiBioGen consortium	European	18,340	https://mibiogen.gcc.rug.nl/
Outcome					
Cholecystitis	finngen_R8_K11_CHOLECYST	FinnGen	European	305,153	https://r8.finngen.fi/
Vitamin A (Retinol)	PMID: 36635386	GWAS Catalog	European	8,247	https://www.ebi.ac.uk/gwas/studies/GCST90200405
Vitamin B_12_	PMID: 33441150	GWAS Catalog	European	19,415	https://www.ebi.ac.uk/gwas/studies/GCST90012772
Vitamin C	PMID: 33203707	–	European	52,018	https://doi.org/10.2337/dc20-1328
Vitamin D (25-hydroxyvitamin D)	PMID: 33064751	–	European	120,618	https://doi.org/10.1371/journal.pmed.1003394
Vitamin E (Alpha-tocopherol)	PMID: 36635386	GWAS Catalog	European	8,192	https://www.ebi.ac.uk/gwas/studies/GCST90200302

*N*, sample size.

### 2.3. Instrument variable selection

In this study, the selection of instrument variables was guided by relevant MR studies, using a threshold of *p* < 1 × 10^−5^ to preliminarily identify single nucleotide polymorphisms (SNP) loci with statistical significance from the GWAS summary data of 211 gut microbiota. Additionally, linkage disequilibrium coefficient *r*^2^ = 0.001 and a region width of 10,000 kb were set to minimize the impact of genetic pleiotropy on the results (29). F-statistics were employed to assess whether the selected instrument variables were weak instruments, with the criterion of *F* > 10 (F=Beta^2^/SE^2^), indicating the absence of weak instrument bias (30). Moreover, the MR-PRESSO test was applied to assess potential horizontal pleiotropy, and any impact from pleiotropy was eliminated by removing outliers. To further evaluate whether individual SNPs are closely associated with cholecystitis and to mitigate potential confounding factors, each SNP’s secondary phenotype was manually checked in PhenoScanner (31).^1^

### 2.4. Mendelian randomization analysis

In this study, three methods, namely MR-Egger regression, Inverse Variance Weighted (IVW) random-effects method, and Weighted Median method, were utilized to validate the causal relationship between 211 gut microbiota and cholecystitis. The Weighted Median method ranks the effect estimates of different instrument variables and obtains the estimate of causal effect by weighted median averaging. This method can handle scenarios where up to half of the instrument variables are unbiased and robust to outliers. The IVW method, a commonly used MR analysis approach and a model averaging method, computes a weighted average of the effect estimates for each genetic variant based on their variances and covariances. It assumes that genetic variants are unbiased instrument variables and share a common causal effect. The IVW method provides an overall estimate of the causal effect. The MR-Egger regression method is primarily employed to address horizontal pleiotropy (32). It allows for the estimation of the causal effect in the presence of horizontal heterogeneity, under the assumption that the genetic variants do not violate other common instrumental variable assumptions. An important assumption of the MR-Egger method is the unbiasedness of the pleiotropy factor, indicating no correlation between the genetic variant’s impact on the outcome variable and its impact on the exposure variable (22). Horizontal pleiotropy refers to the situation where genetic variants have effects on the exposure and outcome variables through pathways other than the expected exposure-outcome pathway. The main analytical method employed in this study is the IVW method, with the other methods serving as supplementary analyses to the IVW method.

### 2.5. Sensitivity analysis

To ensure the accuracy of our research findings and address potential biases, we conducted a series of sensitivity analyses and quality control measures. Firstly, LDSC analysis was performed to address the issue of sample overlap among the selected multiple mediators. This involved assessing the genetic correlations between the included mediators and conducting Bonferroni correction on the *p*-values of the MR analyses for multiple circulating vitamin levels and cholecystitis. Subsequently, sensitivity analyses were conducted. First, Cochrane’s Q test was employed to evaluate heterogeneity between instrument variables. This test compares observed effect sizes with expected effect sizes and identifies heterogeneity when the significance level is below *p* < 0.05 (33). Next, MR-Egger regression was used to examine the presence of pleiotropy. MR-Egger regression estimates the pleiotropic intercept, representing the average pleiotropic effect of all genetic variants (34). If the *p*-value of the intercept is greater than 0.05, pleiotropy can be ruled out. Additionally, to further evaluate and correct for potential pleiotropy, the MR-PRESSO method was utilized. MR-PRESSO identifies and corrects for outliers in the instrument variables, reducing the impact of horizontal pleiotropy on causal estimates. By adjusting for potential pleiotropic effects, this method provides more reliable and robust estimates of causal effects (35).

Although traditional MR-Egger regression and MR-PRESSO methods adequately address the issue of unrelated horizontal pleiotropy, they do not consider the potential bias caused by related horizontal pleiotropy. Incorporating both related and unrelated horizontal pleiotropy can offer more reliable estimates of causal effects. Therefore, in this study, we applied the latest cML-MA method to account for the impact of related horizontal pleiotropy on the results (36).

### 2.6. Reverse Mendelian randomization

In this study, reverse MR analysis will be conducted using selected gut microbiota that are causally related to cholecystitis and circulating vitamin levels. The aim is to eliminate the interference of reverse causality and ensure the reliability of our research findings.

All analyses were performed using the software packages MRcML (version 0.0.0.9), TwoSampleMR (version 0.5.6), and MR-PRESSO (version 1.0) in R version 4.2.1.

## 3. Results

### 3.1. Instrumental variable selection

In this study, we performed instrumental variable selection for the GWAS data of 211 gut microbiota. The F-statistics for all instrumental variables were greater than 10, indicating the absence of weak instrument bias in our analysis. The instrumental variables for different circulating vitamins used in the MR analysis are presented in Table 2. The instrumental variables used for MR analysis of gut microbiota are presented in Supplementary Tables S1, S2, respectively.

**Table 2:** The instrumental variables for different circulating vitamins used in the MR analysis.

Exposure	SNP	EA	OA	beta	se	pval	chr	F
Vitamin A	rs1667226	T	A	0.101	0.015	5.74E-11	18	45.34
Vitamin A	rs1883711	C	G	−0.278	0.045	9.73E-10	20	38.16
Vitamin B_12_	rs1131603	C	T	0.132	0.024	4.18E-08	22	30.25
Vitamin B_12_	rs12780845	G	A	0.066	0.011	7.69E-10	10	36.00
Vitamin B_12_	rs1801222	A	G	−0.090	0.010	5.58E-18	10	81.00
Vitamin B_12_	rs4458686	C	T	−0.070	0.010	2.23E-11	6	49.00
Vitamin B_12_	rs526934	G	A	−0.084	0.011	6.92E-14	11	58.31
Vitamin C	rs10051765	C	T	0.039	0.007	3.64E−9	5	31.04
Vitamin C	rs10136000	A	G	0.04	0.007	1.33E−8	14	32.65
Vitamin C	rs117885456	A	G	0.078	0.012	1.70E−11	12	42.25
Vitamin C	rs174547	C	T	0.036	0.007	3.84E−8	11	26.45
Vitamin C	rs2559850	A	G	0.058	0.006	6.30E−20	12	93.44
Vitamin C	rs33972313	C	T	0.36	0.018	4.61E−90	5	400.0
Vitamin C	rs6693447	T	G	0.039	0.006	6.25E−10	1	42.25
Vitamin C	rs7740812	G	A	0.038	0.006	1.88E−9	6	40.11
Vitamin C	rs9895661	T	C	0.063	0.008	1.05E−14	17	62.02
Vitamin D	rs11203339	C	T	0.012	0.002	4.64E−08	1	36.00
Vitamin D	rs116970203	G	A	0.381	0.022	1.19E−64	11	299.92
Vitamin D	rs12785878	T	G	0.044	0.002	5.60E−87	11	484.00
Vitamin D	rs17216707	T	C	0.03	0.003	1.61E−29	20	100.00
Vitamin D	rs17862870	G	A	0.021	0.004	5.57E−09	2	27.56
Vitamin D	rs3213737	G	A	0.019	0.002	2.05E−19	12	90.25
Vitamin D	rs3755967	C	T	0.106	0.002	2.48E−465	4	2809.00
Vitamin D	rs9304669	T	C	0.052	0.010	4.53E−08	19	27.04
Vitamin E	rs631106	A	C	−0.096	0.016	4.12E-09	1	36.00

EA, effective alleles; OA, other alleles; chr, chromosome.

### 3.2. Causal associations of gut microbiota with cholecystitis

**Figure 2:**
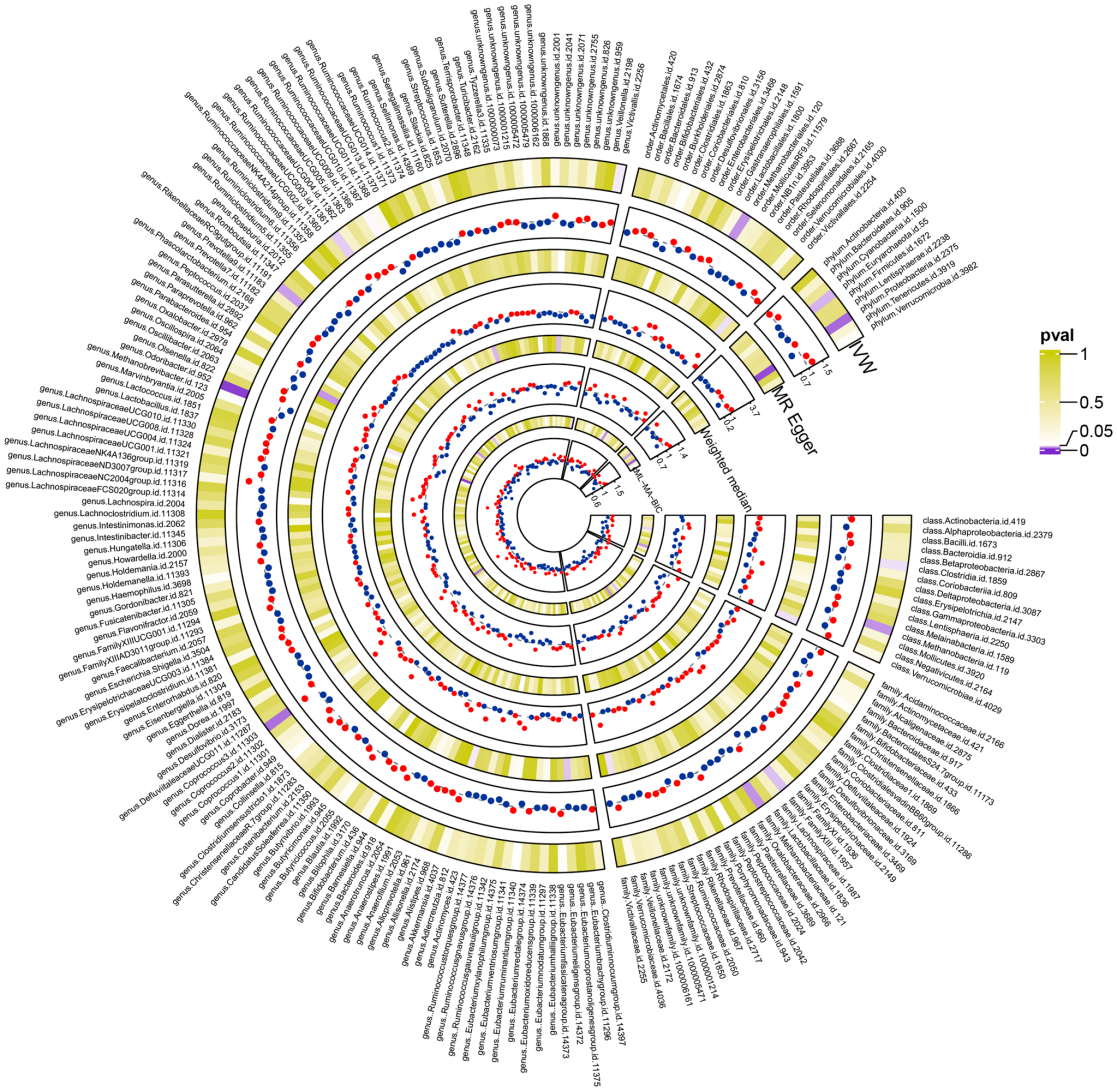
The complete results plot of the Mendelian randomization analysis between 211 gut microbiota taxa and gallbladder inflammation (the figure displays the results of three major Mendelian randomization analyses involving 211 taxa of gut microbiota and gallbladder inflammation. It also presents the results of the cML-MA analysis conducted to mitigate the effects of multicollinearity at the relevant levels).

**Figure 3:**
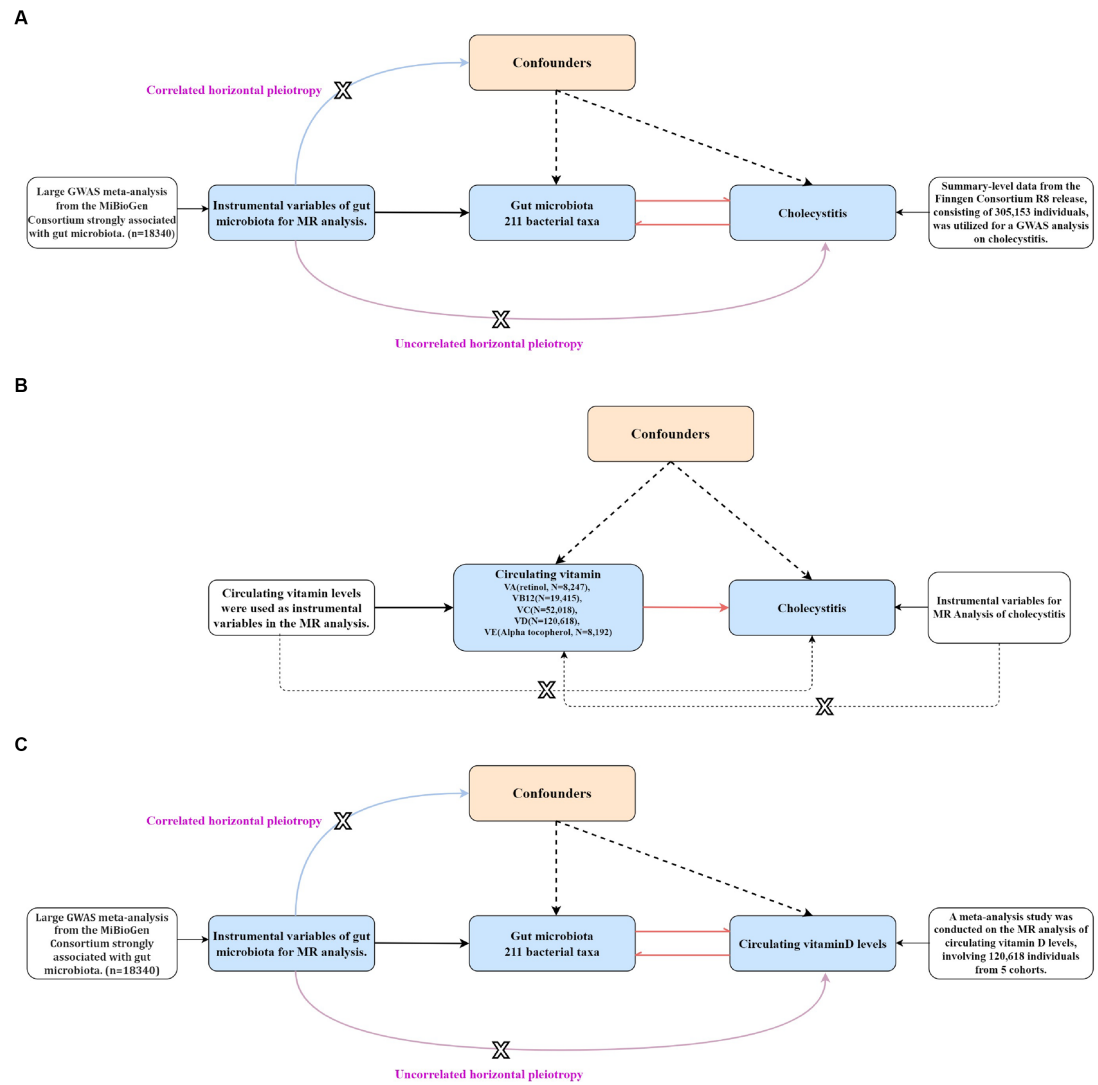
The step-by-step flowchart of the Mendelian randomization analysis (the figure specifically presents the basic assumptions and principles of all Mendelian randomizations conducted in this study and clearly indicates the level of pleiotropy of genes that are correlated and unrelated). **(A)** A bidirectional Mendelian randomization analysis was conducted to assess the causal relationship between gut microbiota and cholecystitis. **(B)** A univariable Mendelian randomization analysis was performed to assess the causal relationship between different circulating vitamin levels and cholecystitis. **(C)** A bidirectional Mendelian randomization analysis was conducted to assess the causal relationship between gut microbiota and circulating vitamin D levels.

Using the IVW method, we identified 13 gut microbial taxa that may be causally associated with cholecystitis. Among them, *RuminococcaceaeUCG003* showed a significant association (OR = 1.25, 95%CI = 1.01–1.54, *p* = 0.04). This result was supported by sensitivity analyses using the weighted median and MR-PRESSO methods. No evidence of horizontal pleiotropy was observed, and the cML-MA test indicated the absence of vertical pleiotropy (*p* = 0.036). The univariable MR analysis yielded an effect size (β_1_) of 0.22 for the causal association between *RuminococcaceaeUCG003* and cholecystitis. The results of other gut microbial taxa possibly associated with cholecystitis risk are presented in Table 3. The overall MR analysis of gut microbiota and cholecystitis is depicted in Figure 2, and the conceptual framework of this analysis is shown in Figure 3A.

**Table 3:** The sensitivity analysis results regarding the association between gut microbiota and cholecystitis.

Outcome	Bacteria taxa (Exposure)	Heterogeneity test		MR-Egger		MR-PRESSO		cML-MA
		Cochran’s Q	*p*	Intercept value	*p*	RSSobs	*p*	*p*
Cholecystitis	Clostridia	9.20	0.82	0.043	0.21	10.69	0.81	0.04
Cholecystitis	Methanobacteria	12.01	0.28	0.029	0.53	14.36	0.30	0.02
Cholecystitis	Family XIII	8.65	0.37	−0.039	0.40	11.02	0.40	0.04
Cholecystitis	Methanobacteriaceae	12.01	0.28	0.029	0.53	14.36	0.33	0.02
Cholecystitis	Coprococcus3	6.01	0.54	−0.027	0.62	8.02	0.54	0.02
Cholecystitis	Odoribacter	3.84	0.80	−0.039	0.31	5.09	0.79	0.01
Cholecystitis	RikenellaceaeRC9gutgroup	8.96	0.63	−0.075	0.18	10.64	0.63	0.03
Cholecystitis	RuminococcaceaeUCG003	9.96	0.70	0.049	0.08	12.01	0.68	0.04
Cholecystitis	RuminococcaceaeUCG010	1.52	0.98	0.003	0.92	1.92	0.99	0.04
Cholecystitis	Victivallis	11.02	0.44	0.034	0.54	13.08	0.50	0.04
Cholecystitis	Methanobacteriales	12.01	0.28	0.029	0.53	14.36	0.32	0.02
Cholecystitis	Euryarchaeota	16.76	0.08	0.020	0.72	20.03	0.11	0.03
Cholecystitis	Proteobacteria	12.63	0.48	0.057	0.03	15.09	0.46	0.01

Subsequently, we performed a reverse MR analysis using the 13 selected gut microbial taxa that were genetically associated with the risk of cholecystitis. However, no evidence of bidirectional causality between gut microbiota and cholecystitis was found. The instrumental variables used for the reverse MR analysis are listed in Supplementary Table S6. The MR analysis results of gut microbiota and cholecystitis are presented in Supplementary Table S4.

### 3.3. Causal associations of circulating vitamin levels with cholecystitis

**Figure 4:**
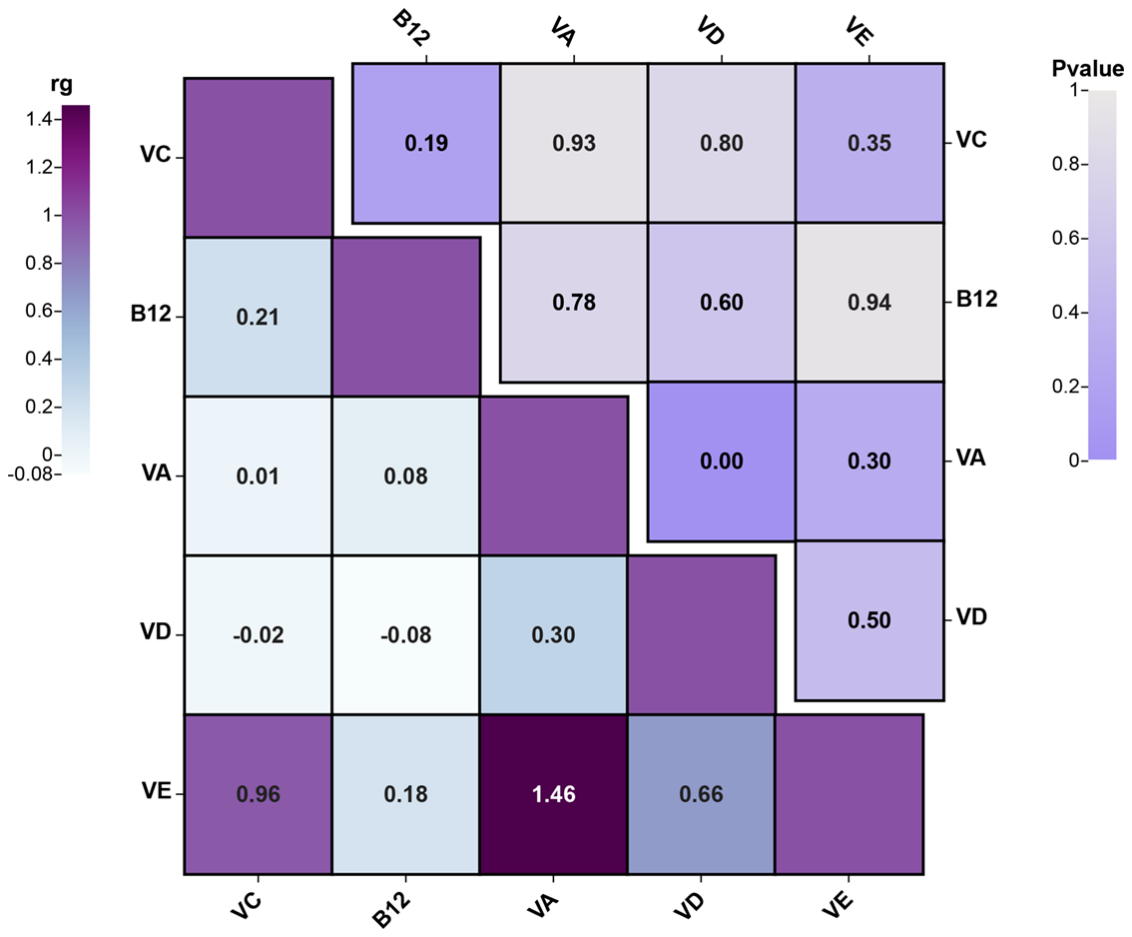
The results plot of LDSC analysis for genetic correlation between different circulating vitamin levels’ GWAS summary data (the figure showcases the genetic correlations among different circulating vitamin levels and combines the *p*-values from the LDSC analysis results. rg, genetic correlation).

Before conducting MR analysis, we first assessed the genetic correlations of the five circulating vitamin levels using LDSC analysis (Figure 4). The analysis revealed a genetic correlation between circulating vitamin A and vitamin D levels (rg = 0.3, *p* = 0.004). Subsequently, we performed univariable MR analyses for each of the five circulating vitamin levels with cholecystitis (Supplementary Table S3). Only circulating vitamin D levels showed a significant causal association with cholecystitis after Bonferroni correction (OR = 0.67, 95%CI = 0.49–0.90, *p* = 0.008, P_adjust_ = 0.04). As no causal association was observed between circulating vitamin A levels and cholecystitis, and the genetic correlation between circulating vitamin A and vitamin D levels did not introduce bias in our results, we did not conduct a multivariable MR analysis using Bayesian model averaging and only included circulating vitamin D level as the mediator in this study. The univariable MR analysis yielded an effect size (β_3_) of −0.41 for the causal association between circulating vitamin D level and cholecystitis. The overall MR analysis of circulating vitamin levels and cholecystitis is depicted in Figure 2, and the conceptual framework of this analysis is shown in Figure 3B.

### 3.4. Causal associations of gut microbiota with circulating vitamin D levels

After identifying the mediator, circulating vitamin D levels, we performed IVW analysis and identified 15 gut microbial taxa that may be causally associated with circulating vitamin D levels. Notably, the results for *RuminococcaceaeUCG003* were also significant (OR = 0.97, 95% CI = 0.944–0.998, *p* = 0.04). This finding was supported by MR-PRESSO sensitivity analysis, and no evidence of horizontal or vertical pleiotropy was observed (*p* = 0.035) using the cML-MA test. The univariable MR analysis yielded an effect size (β2) of −0.03 for the causal association between *RuminococcaceaeUCG003* and circulating vitamin D levels. The results of other gut microbial taxa that may be associated with circulating vitamin D levels are presented in Supplementary Table S5, and the conceptual framework of this analysis is shown in Figure 3C.

Similarly, we conducted a reverse MR analysis using the 15 selected gut microbial taxa that were genetically associated with circulating vitamin D levels. However, no evidence of bidirectional causality between specific gut microbial taxa and circulating vitamin D levels was found.

### 3.5. Mediation effect of circulating vitamin D levels in the causal association between *RuminococcaceaeUCG003* and cholecystitis

**Figure 5:**
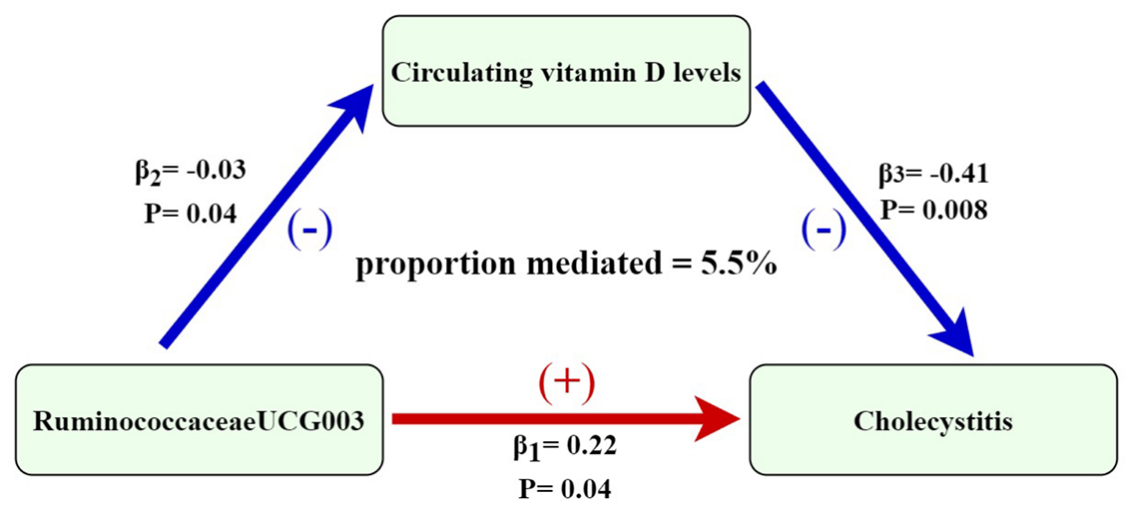
Mediation effect of circulating vitamin D levels in the causal association between RuminococcaceaeUCG003 and cholecystitis [the figure shows that the risk of cholecystitis is increased when the abundance of RuminococcaceaeUCG003 increases, with an effect size of 0.22. 25-hydroxyvitamin D levels decreased with increasing abundance of RuminococcaceaeUCG003, with an effect value of −0.03. The risk of cholecystitis increased with a decrease in circulating vitamin D levels, with an effect size of −0.41. The association between gut microbiota and risk of cholecystitis is mediated by circulating vitamin D levels (proportion mediated = 5.5%)].

Through two-step MR analysis, we calculated a potential mediation effect of circulating vitamin D levels (proportion mediated = 5.5%) in the causal association between *RuminococcaceaeUCG003* and the risk of cholecystitis. This implies that an increase of one standard deviation in *RuminococcaceaeUCG003* abundance would result in a 25% increase in cholecystitis risk, accompanied by a 3% decrease in circulating vitamin D levels. Conversely, a one standard deviation decrease in circulating vitamin D levels would increase the risk of cholecystitis by 33%. The mediation effect is illustrated in Figure 5. In addition, scatter plots and leave-one-out analyses of the main results from the two-sample MR analysis conducted in this study are presented in Supplementary Figure S1.

## 4. Discussion

This study employed large-scale GWAS summary data and applied a two-sample MR approach to investigate the association between gut microbiota, serum vitamin levels, and cholecystitis. Our findings revealed a positive correlation between an increase in the abundance of gut microbiota *RuminococcaceaeUCG003* and an elevated risk of cholecystitis. Additionally, we observed that the increased risk of cholecystitis might be mediated by a decline in circulating vitamin D levels (proportion mediated = 5.5%).

Firstly, we observed a significant correlation between gut microbiota dysbiosis and the risk of cholecystitis, consistent with prior research. Studies suggest that bacteria can colonize the gallbladder either through the duodenal-ampullary complex or by hematogenous spread to the liver, eventually leading to the formation of a microbial environment in the gallbladder (37). For instance, Hu et al., using ITS and 16S rRNA gene high-throughput sequencing, studied the fungal and bacterial spectra in bile and gallstone samples from cholelithiasis patients, identifying the presence of *Ruminococcus* genus and other gallbladder microbiota consistent with our findings (38). Gut microbiota dysbiosis may promote the development of cholecystitis through multiple mechanisms. Firstly, it may trigger an inflammatory response within the gallbladder, leading to cholecystitis (39). Furthermore, a meta-analysis has shown a positive association between *Helicobacter pylori* infection and chronic cholecystitis and cholelithiasis (OR = 3.022, 95% CI: 1.897–4.815, I^2^ = 20.1%), this could be due to *H. pylori* infection causing chronic inflammation of the biliary mucosa, leading to impaired bile acid secretion and increased risk of gallstone formation (11). Secondly, gut microbiota dysbiosis may affect the metabolism of bile acids, leading to changes in bile acid composition and function, thus promoting the formation of gallstones and the development of cholecystitis (40).

Recently, research on the relationship between gut microbiota and circulating vitamin levels has become a hot topic. Gut microbiota metabolism can influence the synthesis, absorption, and metabolism of vitamins. For example, LeBlanc et al. proposed that B vitamins can be synthesized through lactate and potentially by bifidobacteria present in the gut (41). On the other hand, Traber et al. suggested that dietary overnutrition could lead to an increased abundance of gram-negative bacteria in the gut, causing increased inflammation and compromised gut function, subsequently affecting the status of antioxidant vitamins (such as vitamin C and vitamin E) (42). Additionally, some studies have indicated a high expression of vitamin D receptors in the gastrointestinal tract, but the relationship between gut microbiota and vitamin D is not yet well-established (43). Hence, we aimed to elucidate the mediating role of different circulating vitamin levels in the association between gut microbiota and cholecystitis. The causal relationship between serum vitamin levels and gut microbiota, influencing the occurrence of cholecystitis, may involve various mechanisms. Firstly, dysregulated gut microbiota may reduce vitamin A levels, and the metabolite all-trans retinoic acid plays a crucial role in mucosal immune response, with immune dysregulation contributing to the development of cholecystitis (44). Secondly, the reduced levels of vitamins such as vitamin C and vitamin E may impair the antioxidant defense system, increase oxidative stress in the gallbladder, and thus promote the development of cholecystitis (45). Our primary finding was that circulating vitamin D levels mediated the causative relationship between *RuminococcaceaeUCG003* and cholecystitis. Research suggests that circulating vitamin D can affect the normal contraction of the gallbladder and bile excretion, with a decline in circulating vitamin D levels leading to bile stasis and consequent cholecystitis (46). For instance, Fangji Yang et al. using NTCP-deficient mice (exhibiting common phenotypes of high choline blood levels, vitamin D deficiency, bone loss, and gallbladder anomalies), observed significant changes in serum bile acid levels. Therefore, there is a close relationship between circulating vitamin D levels and bile acid status. Moreover, as cholecystitis is often associated with bacterial infections, and vitamin D regulates the innate immune system, enhancing the cellular killing of pathogenic microorganisms, some studies have suggested an inverse correlation between serum 25(OH)D levels and infection risk (47). In conclusion, our study revealed complex interactions and potential mechanisms between gut microbiota, serum vitamin levels, and cholecystitis. Important mechanisms may include gut microbiota dysbiosis-induced inflammation, alterations in bile acid metabolism, immune imbalance, compromised antioxidant defense systems, impaired gut barrier function, and gallstone formation. These findings are of significant importance for understanding the potential relationships between gut microbiota, serum vitamin levels, and cholecystitis. Future research should further explore potential therapeutic strategies for preventing and managing cholecystitis by modulating gut microbiota and supplementing vitamin levels.

Moreover, it is essential to acknowledge the limitations of this study. Firstly, MR relies on the fundamental assumption that genetic variations serving as instrumental variables influence the outcome solely through the exposure factor. However, despite our efforts to minimize confounding factors and use the cML-MA method to address pleiotropy, there may still be unmeasured confounding factors. Secondly, our study primarily focused on European populations, and the generalizability of our findings to other populations may be limited. We acknowledge that other potential mediators, aside from circulating vitamin levels, could contribute to the observed relationship between gut microbiota and cholecystitis. Future studies could explore these additional mediating factors to provide a more comprehensive understanding of the complex interactions involved. In addition, future research should consider utilizing larger samples of genetic variations in the gut microbiota to conduct a more comprehensive evaluation of our study.

## 5. Conclusion

In summary, our two-sample MR analysis unveiled the causal relationship between circulating vitamin D levels mediating gut microbiota and cholecystitis, along with the underlying mechanisms. Future research should further explore potential therapeutic strategies by modulating gut microbiota and supplementing vitamin levels to prevent and mitigate the risk of cholecystitis.

## Data availability statement

The datasets presented in this study can be found in online repositories. The names of the repository/repositories and accession number(s) can be found in the article/Supplementary material.

## Ethics statement

Ethical approval was not required for the study involving humans in accordance with the local legislation and institutional requirements.

## Author contributions

CM: Writing – original draft, Writing – review & editing. LX: Writing – original draft, Writing – review & editing. XX: Software, Writing – original draft, Writing – review & editing. SH: Writing – original draft. JL: Investigation, Writing – review & editing. KC: Writing – original draft, Writing – review & editing.

## Footnotes

^1^ http://www.phenoscanner.medschl.cam.ac.uk/
