# Supplementary material for: Circulating vitamin levels mediate the causal relationship between gut microbiota and cholecystitis: a two-step bidirectional Mendelian randomization study
Source: Front Nutr. 2023 Sep 26;10:1268893. doi: 10.3389/fnut.2023.1268893 (PMC10562588; doi:10.3389/fnut.2023.1268893)
Supplement: Supplementary file 1 [file Data_Sheet_1.docx]

Supplementary Material

**Circulating Vitamin Levels mediate the Causal relationship between gut microbiota and Cholecystitis: A Two-step Bidirectional Mendelian Randomization Study**

**Changhong Miao^1,2^†, Lu Xiao^1,2^†, Xinyi Xu^1,2^†, Shuoxuan Huang^1,2^, Jiajin Liu^1,2^, Kuang Chen^1,2,*^**

^1^First Teaching Hospital of Tianjin University of Traditional Chinese Medicine, Tianjin, China

^2^National Clinical Research Center for Chinese Medicine Acupuncture and Moxibustion, Tianjin, China

†Changhong Miao, Lu Xiao, and Xinyi Xu contributed equally to this work and are co-first authors.

***Corresponding Author:**

Kuang Chen

First Teaching Hospital of Tianjin University of Traditional Chinese Medicine, Tianjin, China

1. mail: [18622150281@163.com](mailto:18622150281@163.com)

# 1 Supplementary Tables

**Table S1** Instrumental variable information used for Mendelian randomization on the association between gut microbiota and circulating vitamin D levels.

| Gut microbiota--Vitamin D | | | | | | | |
| --- | --- | --- | --- | --- | --- | --- | --- |
| Exposure | SNP | EA | OA | beta | se | pval | F |
| Coriobacteriia | rs11073596 | T | G | 0.05 | 0.01 | 8.14E-06 | 25 |
|  | rs11250875 | T | C | 0.06 | 0.01 | 4.83E-06 | 36 |
|  | rs11656361 | A | C | 0.08 | 0.02 | 8.02E-06 | 16 |
|  | rs12974142 | G | A | 0.08 | 0.02 | 8.51E-06 | 16 |
|  | rs13307134 | C | T | 0.06 | 0.01 | 7.80E-06 | 36 |
|  | rs1397793 | G | A | -0.05 | 0.01 | 9.77E-06 | 25 |
|  | rs1816223 | A | G | -0.06 | 0.01 | 4.84E-06 | 36 |
|  | rs240104 | T | C | -0.06 | 0.01 | 1.52E-06 | 36 |
|  | rs2442778 | G | A | -0.12 | 0.03 | 9.03E-06 | 16 |
|  | rs3025411 | A | G | 0.10 | 0.02 | 8.27E-06 | 25 |
|  | rs34739816 | G | T | 0.10 | 0.02 | 3.88E-06 | 25 |
|  | rs67561917 | A | G | -0.07 | 0.02 | 5.39E-06 | 12.25 |
|  | rs719099 | A | G | 0.08 | 0.02 | 5.43E-07 | 16 |
|  | rs8010111 | G | A | -0.10 | 0.02 | 6.90E-06 | 25 |
| Coriobacteriaceae | rs11073596 | T | G | 0.05 | 0.01 | 8.14E-06 | 25 |
|  | rs11250875 | T | C | 0.06 | 0.01 | 4.83E-06 | 36 |
|  | rs11656361 | A | C | 0.08 | 0.02 | 8.02E-06 | 16 |
|  | rs12974142 | G | A | 0.08 | 0.02 | 8.51E-06 | 16 |
|  | rs13307134 | C | T | 0.06 | 0.01 | 7.80E-06 | 36 |
|  | rs1397793 | G | A | -0.05 | 0.01 | 9.77E-06 | 25 |
|  | rs1816223 | A | G | -0.06 | 0.01 | 4.84E-06 | 36 |
|  | rs240104 | T | C | -0.06 | 0.01 | 1.52E-06 | 36 |
|  | rs2442778 | G | A | -0.11 | 0.03 | 9.03E-06 | 13.44 |
|  | rs3025411 | A | G | 0.10 | 0.02 | 8.27E-06 | 25 |
|  | rs34739816 | G | T | 0.10 | 0.02 | 3.88E-06 | 25 |
|  | rs67561917 | A | G | -0.07 | 0.02 | 5.39E-06 | 12.25 |
|  | rs719099 | A | G | 0.08 | 0.02 | 5.43E-07 | 16 |
|  | rs8010111 | G | A | -0.10 | 0.02 | 6.90E-06 | 25 |
| Lactobacillaceae | rs11674854 | C | T | -0.08 | 0.02 | 2.60E-06 | 16 |
|  | rs1530559 | G | A | 0.08 | 0.02 | 9.65E-06 | 16 |
|  | rs16861661 | G | A | -0.19 | 0.04 | 2.70E-07 | 22.56 |
|  | rs62314653 | C | A | 0.18 | 0.04 | 6.59E-06 | 20.25 |
|  | rs74599091 | A | G | 0.19 | 0.04 | 7.70E-06 | 22.56 |
|  | rs768253 | T | G | -0.08 | 0.02 | 3.61E-06 | 16 |
|  | rs77478751 | A | G | -0.22 | 0.05 | 5.96E-06 | 19.36 |
|  | rs921925 | A | C | 0.10 | 0.02 | 5.77E-07 | 25 |
|  | rs9345899 | A | G | -0.12 | 0.03 | 9.45E-06 | 16 |
| Prevotellaceae | rs12057990 | C | T | 0.06 | 0.01 | 8.97E-06 | 36 |
|  | rs12118202 | T | C | -0.08 | 0.01 | 5.54E-07 | 64 |
|  | rs13069367 | A | C | -0.05 | 0.01 | 7.39E-06 | 25 |
|  | rs148376875 | T | G | 0.08 | 0.02 | 2.08E-06 | 16 |
|  | rs2206482 | T | G | -0.06 | 0.01 | 1.30E-06 | 36 |
|  | rs2278540 | G | A | 0.06 | 0.01 | 8.44E-06 | 36 |
|  | rs34660375 | A | G | -0.08 | 0.02 | 7.40E-06 | 16 |
|  | rs3758087 | T | C | 0.06 | 0.01 | 8.61E-06 | 36 |
|  | rs3860225 | A | G | 0.08 | 0.02 | 5.50E-07 | 16 |
|  | rs4493272 | T | C | -0.06 | 0.01 | 3.02E-07 | 36 |
|  | rs4685827 | T | C | -0.07 | 0.01 | 2.77E-06 | 49 |
|  | rs7252711 | A | G | -0.07 | 0.02 | 5.57E-06 | 12.25 |
|  | rs7975087 | C | A | -0.06 | 0.01 | 7.59E-06 | 36 |
|  | rs912860 | G | A | -0.22 | 0.05 | 9.30E-07 | 19.36 |
|  | rs9586501 | G | A | 0.06 | 0.01 | 2.59E-06 | 36 |
|  | rs9958960 | G | A | -0.09 | 0.02 | 1.06E-07 | 20.25 |
| Anaerotruncus | rs10150232 | A | G | 0.06 | 0.01 | 6.68E-06 | 36 |
|  | rs11018566 | A | G | -0.16 | 0.04 | 6.14E-06 | 16 |
|  | rs115414803 | A | C | -0.14 | 0.03 | 6.83E-06 | 21.78 |
|  | rs1272208 | G | T | -0.06 | 0.01 | 4.28E-06 | 36 |
|  | rs1431492 | C | T | -0.07 | 0.01 | 7.36E-06 | 49 |
|  | rs17734739 | T | C | 0.07 | 0.01 | 7.43E-06 | 49 |
|  | rs34449434 | A | C | -0.05 | 0.01 | 9.85E-06 | 25 |
|  | rs4669806 | G | T | 0.06 | 0.01 | 2.42E-06 | 36 |
|  | rs6494922 | A | G | 0.09 | 0.02 | 6.62E-06 | 20.25 |
|  | rs6563550 | T | C | 0.09 | 0.02 | 2.35E-07 | 20.25 |
|  | rs7155595 | C | A | 0.05 | 0.01 | 7.55E-06 | 25 |
|  | rs8005030 | C | T | 0.06 | 0.01 | 2.28E-06 | 36 |
|  | rs9347879 | T | C | 0.05 | 0.01 | 4.22E-06 | 25 |
| Escherichia | rs112767262 | T | C | 0.07 | 0.02 | 8.21E-06 | 12.25 |
|  | rs113127095 | A | G | 0.15 | 0.03 | 3.33E-06 | 25 |
|  | rs113513883 | A | G | 0.17 | 0.04 | 5.28E-06 | 18.06 |
|  | rs1154904 | A | G | -0.06 | 0.01 | 3.04E-06 | 36 |
|  | rs118526 | C | A | -0.06 | 0.01 | 8.00E-06 | 36 |
|  | rs2798105 | A | G | -0.10 | 0.02 | 8.25E-06 | 25 |
|  | rs4731451 | G | A | -0.06 | 0.01 | 7.47E-06 | 36 |
|  | rs57024273 | T | C | 0.06 | 0.01 | 9.70E-06 | 36 |
|  | rs592299 | T | C | -0.06 | 0.01 | 4.77E-06 | 36 |
|  | rs73208162 | A | G | -0.11 | 0.02 | 2.19E-06 | 30.25 |
| LachnospiraceaeND3007group | rs2861203 | G | A | 0.06 | 0.01 | 7.37E-06 | 36 |
|  | rs72776675 | T | C | -0.06 | 0.01 | 8.72E-06 | 36 |
|  | rs9932954 | A | G | -0.06 | 0.01 | 1.25E-06 | 36 |
| Lactobacillus | rs11674854 | C | T | -0.09 | 0.02 | 1.59E-06 | 20.25 |
|  | rs12693845 | C | T | -0.08 | 0.02 | 8.96E-06 | 16 |
|  | rs1530559 | G | A | 0.08 | 0.02 | 4.93E-06 | 16 |
|  | rs16861661 | G | A | -0.18 | 0.04 | 1.28E-06 | 20.25 |
|  | rs62314653 | C | A | 0.19 | 0.04 | 2.24E-06 | 22.56 |
|  | rs7399658 | G | A | -0.11 | 0.02 | 3.12E-06 | 30.25 |
|  | rs75127669 | C | A | 0.14 | 0.03 | 6.83E-06 | 21.78 |
|  | rs768253 | T | G | -0.08 | 0.02 | 4.25E-06 | 16 |
|  | rs77478751 | A | G | -0.22 | 0.05 | 7.33E-06 | 19.36 |
|  | rs921925 | A | C | 0.10 | 0.02 | 9.72E-07 | 25 |
| Oscillibacter | rs11627628 | T | C | 0.14 | 0.03 | 1.01E-06 | 21.78 |
|  | rs11990279 | T | C | -0.08 | 0.02 | 4.94E-06 | 16 |
|  | rs12649930 | T | G | 0.12 | 0.03 | 4.09E-06 | 16 |
|  | rs133832 | A | C | -0.08 | 0.02 | 1.15E-06 | 16 |
|  | rs16866406 | A | G | 0.10 | 0.02 | 3.08E-06 | 25 |
|  | rs16934185 | A | G | -0.13 | 0.03 | 4.38E-06 | 18.78 |
|  | rs234108 | A | G | 0.07 | 0.02 | 9.16E-07 | 12.25 |
|  | rs36095275 | C | T | -0.08 | 0.02 | 1.40E-06 | 16 |
|  | rs4506202 | A | G | -0.07 | 0.02 | 3.21E-06 | 12.25 |
|  | rs61883564 | A | G | -0.10 | 0.02 | 3.39E-06 | 25 |
|  | rs62206502 | C | A | -0.07 | 0.02 | 6.60E-06 | 12.25 |
|  | rs75453768 | G | T | 0.12 | 0.03 | 5.35E-06 | 16 |
|  | rs761240 | T | G | -0.18 | 0.04 | 2.04E-06 | 20.25 |
|  | rs9393920 | A | G | -0.07 | 0.02 | 9.92E-07 | 12.25 |
| Parasutterella | rs10899911 | A | G | -0.07 | 0.01 | 1.15E-06 | 49 |
|  | rs11715853 | G | A | -0.07 | 0.01 | 6.23E-06 | 49 |
|  | rs2090816 | A | C | 0.08 | 0.02 | 2.90E-06 | 16 |
|  | rs2387977 | T | C | -0.07 | 0.01 | 5.38E-07 | 49 |
|  | rs35055552 | T | C | 0.11 | 0.02 | 3.35E-06 | 30.25 |
|  | rs55877868 | A | C | -0.10 | 0.02 | 2.87E-06 | 25 |
|  | rs62273907 | A | G | 0.23 | 0.05 | 5.88E-06 | 21.16 |
|  | rs6809952 | G | A | -0.07 | 0.02 | 8.13E-06 | 12.25 |
|  | rs6828768 | C | T | 0.06 | 0.01 | 1.78E-06 | 36 |
|  | rs7303158 | C | T | 0.06 | 0.01 | 1.33E-06 | 36 |
|  | rs7311004 | T | C | -0.06 | 0.01 | 5.92E-06 | 36 |
|  | rs7572229 | G | A | 0.06 | 0.01 | 6.32E-07 | 36 |
|  | rs78383039 | T | C | -0.15 | 0.023 | 1.57E-06 | 42.53 |
|  | rs8039785 | T | G | 0.06 | 0.01 | 3.62E-06 | 36 |
|  | rs823424 | G | A | -0.07 | 0.02 | 4.95E-06 | 12.25 |
| RuminococcaceaeUCG003 | rs10490280 | C | T | -0.07 | 0.01 | 4.16E-06 | 49 |
|  | rs11243416 | T | C | -0.10 | 0.02 | 1.67E-06 | 25 |
|  | rs11613919 | G | T | 0.07 | 0.02 | 1.63E-06 | 12.25 |
|  | rs16959793 | A | C | -0.06 | 0.01 | 2.22E-06 | 36 |
|  | rs2523124 | T | C | -0.05 | 0.01 | 5.78E-06 | 25 |
|  | rs3013089 | G | A | -0.06 | 0.01 | 4.38E-06 | 36 |
|  | rs4452755 | A | C | -0.06 | 0.01 | 3.29E-06 | 36 |
|  | rs4532474 | G | A | 0.08 | 0.02 | 4.82E-06 | 16 |
|  | rs646327 | G | A | 0.06 | 0.01 | 7.83E-07 | 36 |
|  | rs6759615 | A | G | 0.10 | 0.02 | 7.86E-07 | 25 |
|  | rs73341549 | T | C | -0.17 | 0.03 | 1.51E-07 | 32.11 |
|  | rs78720113 | A | G | -0.12 | 0.02 | 7.59E-06 | 36 |
| RuminococcaceaeUCG013 | rs11581881 | C | T | 0.07 | 0.01 | 4.73E-06 | 49 |
|  | rs12189346 | G | A | 0.07 | 0.01 | 1.68E-06 | 49 |
|  | rs12336782 | T | C | -0.09 | 0.02 | 8.60E-06 | 20.25 |
|  | rs12485353 | G | A | -0.06 | 0.01 | 4.19E-06 | 36 |
|  | rs12781711 | C | T | -0.07 | 0.01 | 2.55E-08 | 49 |
|  | rs16918863 | A | C | 0.11 | 0.02 | 4.16E-06 | 30.25 |
|  | rs2730183 | G | A | -0.05 | 0.01 | 8.44E-06 | 25 |
|  | rs4385846 | G | T | 0.06 | 0.01 | 6.46E-06 | 36 |
|  | rs75088940 | T | C | -0.09 | 0.02 | 2.55E-06 | 20.25 |
|  | rs76973485 | G | T | 0.19 | 0.04 | 3.35E-06 | 22.56 |
|  | rs7784330 | G | A | -0.05 | 0.01 | 8.16E-06 | 25 |
|  | rs9313055 | T | C | 0.11 | 0.02 | 9.55E-06 | 30.25 |
| Streptococcus | rs10028567 | C | T | -0.10 | 0.02 | 7.30E-06 | 25 |
|  | rs10448310 | A | G | -0.05 | 0.01 | 3.31E-06 | 25 |
|  | rs11110281 | T | C | -0.14 | 0.02 | 2.58E-09 | 49 |
|  | rs11720390 | G | A | 0.11 | 0.02 | 3.59E-06 | 30.25 |
|  | rs11764382 | A | G | -0.07 | 0.01 | 1.29E-06 | 49 |
|  | rs17708276 | A | G | -0.08 | 0.02 | 3.04E-06 | 16 |
|  | rs1918540 | G | A | 0.06 | 0.01 | 2.44E-06 | 36 |
|  | rs2370083 | G | T | -0.08 | 0.02 | 9.75E-06 | 16 |
|  | rs4968759 | A | G | -0.05 | 0.01 | 3.78E-06 | 25 |
|  | rs57646748 | G | A | -0.09 | 0.02 | 5.48E-06 | 20.25 |
|  | rs6806351 | T | C | -0.06 | 0.01 | 4.94E-06 | 36 |
|  | rs71481756 | T | G | 0.09 | 0.02 | 6.51E-06 | 20.25 |
|  | rs72739637 | A | G | 0.10 | 0.02 | 1.03E-06 | 25 |
|  | rs7916711 | A | G | 0.10 | 0.02 | 2.72E-06 | 25 |
|  | rs9903102 | C | A | -0.07 | 0.02 | 4.18E-06 | 12.25 |
| unknowngenus | rs10200320 | C | T | 0.06 | 0.01 | 5.66E-06 | 36 |
|  | rs11195523 | A | C | 0.07 | 0.01 | 2.40E-06 | 49 |
|  | rs11684166 | A | G | -0.08 | 0.02 | 3.49E-06 | 16 |
|  | rs11809762 | G | A | -0.09 | 0.02 | 1.68E-06 | 20.25 |
|  | rs11904514 | G | A | -0.11 | 0.02 | 7.90E-06 | 30.25 |
|  | rs12147596 | C | T | -0.07 | 0.01 | 2.86E-07 | 49 |
|  | rs1455639 | G | A | 0.08 | 0.01 | 7.84E-06 | 64 |
|  | rs16823675 | C | T | -0.08 | 0.01 | 2.33E-07 | 64 |
|  | rs17086536 | C | A | -0.10 | 0.02 | 3.36E-06 | 25 |
|  | rs2939766 | A | G | -0.06 | 0.01 | 7.01E-06 | 36 |
|  | rs34985298 | G | A | -0.06 | 0.01 | 8.34E-06 | 36 |
|  | rs35740166 | C | T | -0.11 | 0.02 | 8.40E-07 | 30.25 |
|  | rs4644504 | T | C | -0.10 | 0.02 | 5.82E-06 | 25 |
|  | rs6007642 | C | T | -0.08 | 0.02 | 9.96E-06 | 16 |
|  | rs72700702 | T | C | -0.09 | 0.02 | 1.59E-06 | 20.25 |
|  | rs76532867 | T | C | 0.11 | 0.02 | 2.56E-06 | 30.25 |
| Coriobacteriales | rs11073596 | T | G | 0.05 | 0.01 | 8.14E-06 | 25 |
|  | rs11250875 | T | C | 0.06 | 0.01 | 4.83E-06 | 36 |
|  | rs11656361 | A | C | 0.08 | 0.02 | 8.02E-06 | 16 |
|  | rs12974142 | G | A | 0.08 | 0.02 | 8.51E-06 | 16 |
|  | rs13307134 | C | T | 0.06 | 0.01 | 7.80E-06 | 36 |
|  | rs1397793 | G | A | -0.05 | 0.01 | 9.77E-06 | 25 |
|  | rs1816223 | A | G | -0.06 | 0.01 | 4.84E-06 | 36 |
|  | rs240104 | T | C | -0.06 | 0.01 | 1.52E-06 | 36 |
|  | rs2442778 | G | A | -0.12 | 0.03 | 9.03E-06 | 16 |
|  | rs3025411 | A | G | 0.09 | 0.02 | 8.27E-06 | 20.25 |
|  | rs34739816 | G | T | 0.10 | 0.02 | 3.88E-06 | 25 |
|  | rs67561917 | A | G | -0.07 | 0.02 | 5.39E-06 | 12.25 |
|  | rs719099 | A | G | 0.08 | 0.02 | 5.43E-07 | 16 |
|  | rs8010111 | G | A | -0.10 | 0.02 | 6.90E-06 | 25 |

EA, effective alleles；OA, other alleles

**Table S2** Instrumental variable information used for Mendelian randomization on the association between gut microbiota and Cholecystitis.

| Gut microbiota--Cholecystitis | | | | | | | |
| --- | --- | --- | --- | --- | --- | --- | --- |
| Exposure | SNP | EA | OA | beta | se | pval | F |
| Clostridia | rs10774377 | A | G | 0.05 | 0.01 | 3.24E-06 | 25 |
|  | rs112334273 | G | A | 0.06 | 0.01 | 3.81E-07 | 36 |
|  | rs13105690 | T | C | -0.05 | 0.01 | 8.78E-06 | 25 |
|  | rs13179700 | T | C | 0.05 | 0.01 | 3.37E-06 | 25 |
|  | rs1842454 | G | A | -0.05 | 0.01 | 8.72E-06 | 25 |
|  | rs2273429 | A | G | -0.07 | 0.02 | 4.52E-06 | 12.25 |
|  | rs6797343 | G | T | 0.06 | 0.01 | 9.36E-06 | 36 |
|  | rs6814436 | T | C | 0.07 | 0.02 | 9.65E-07 | 12.25 |
|  | rs6815608 | T | C | 0.10 | 0.02 | 4.02E-07 | 25 |
|  | rs72738886 | T | C | 0.09 | 0.02 | 8.24E-06 | 20.25 |
| Methanobacteria | rs10202904 | T | G | -0.12 | 0.02 | 3.01E-07 | 36 |
|  | rs10424197 | G | A | -0.11 | 0.02 | 9.28E-06 | 30.25 |
|  | rs4257531 | G | A | 0.16 | 0.04 | 7.44E-06 | 16 |
|  | rs62241835 | G | T | -0.20 | 0.04 | 1.63E-06 | 25 |
|  | rs6508769 | T | C | 0.15 | 0.03 | 8.23E-06 | 25 |
|  | rs73068003 | G | T | -0.16 | 0.04 | 8.45E-06 | 16 |
|  | rs73457410 | A | G | 0.22 | 0.04 | 1.41E-06 | 30.25 |
|  | rs75208022 | C | T | -0.23 | 0.05 | 5.92E-06 | 21.16 |
|  | rs894996 | C | A | 0.22 | 0.04 | 1.88E-06 | 30.25 |
| FamilyXIII | rs10404377 | C | A | -0.05 | 0.01 | 6.99E-06 | 25 |
|  | rs118170811 | A | G | 0.15 | 0.03 | 1.80E-06 | 25 |
|  | rs482905 | G | T | 0.06 | 0.01 | 3.72E-06 | 36 |
|  | rs6501525 | A | G | 0.06 | 0.01 | 1.24E-06 | 36 |
|  | rs66753613 | G | A | 0.07 | 0.01 | 8.08E-06 | 49 |
|  | rs6797051 | C | T | -0.08 | 0.02 | 4.89E-06 | 16 |
|  | rs7514702 | T | C | -0.07 | 0.01 | 3.92E-06 | 49 |
| Methanobacteriaceae | rs10202904 | T | G | 0.08 | 0.02 | 3.01E-07 | 16 |
|  | rs10424197 | G | A | -0.07 | 0.01 | 9.28E-06 | 49 |
|  | rs4257531 | G | A | -0.10 | 0.02 | 7.44E-06 | 25 |
|  | rs62241835 | G | T | 0.16 | 0.04 | 1.63E-06 | 16 |
|  | rs6508769 | T | C | 0.09 | 0.02 | 8.23E-06 | 20.25 |
|  | rs73068003 | G | T | -0.09 | 0.02 | 8.45E-06 | 20.25 |
|  | rs73457410 | A | G | -0.04 | 0.01 | 1.41E-06 | 16 |
|  | rs75208022 | C | T | -0.05 | 0.01 | 5.92E-06 | 25 |
|  | rs894996 | C | A | -0.08 | 0.02 | 1.88E-06 | 16 |
| Coprococcus3 | rs10810043 | A | G | 0.05 | 0.01 | 9.27E-06 | 25 |
|  | rs11077359 | T | C | -0.06 | 0.01 | 9.64E-06 | 36 |
|  | rs11080344 | C | T | 0.05 | 0.01 | 4.79E-06 | 25 |
|  | rs13247359 | G | A | 0.05 | 0.01 | 7.33E-06 | 25 |
|  | rs178271 | T | C | 0.15 | 0.03 | 7.81E-07 | 25 |
|  | rs4575475 | G | A | 0.06 | 0.01 | 7.04E-06 | 36 |
|  | rs7521171 | G | A | -0.06 | 0.01 | 4.32E-06 | 36 |
|  | rs8100692 | T | C | 0.06 | 0.01 | 4.16E-07 | 36 |
| Odoribacter | rs10093869 | A | G | -0.06 | 0.01 | 3.67E-06 | 36 |
|  | rs10423795 | C | T | 0.06 | 0.01 | 6.58E-06 | 36 |
|  | rs28417404 | A | G | -0.07 | 0.02 | 3.68E-06 | 12.25 |
|  | rs4793970 | A | G | -0.06 | 0.01 | 6.03E-06 | 36 |
|  | rs6856150 | G | A | 0.09 | 0.02 | 6.06E-06 | 20.25 |
|  | rs74553962 | T | G | 0.12 | 0.03 | 9.49E-06 | 16 |
|  | rs77779484 | G | A | -0.13 | 0.03 | 6.56E-07 | 18.78 |
| RikenellaceaeRC9gutgroup | rs12501673 | A | G | 0.12 | 0.03 | 6.29E-06 | 16 |
|  | rs17032291 | T | C | -0.17 | 0.04 | 6.61E-06 | 18.06 |
|  | rs17582787 | A | G | -0.16 | 0.03 | 3.55E-06 | 28.44 |
|  | rs2074881 | T | C | -0.14 | 0.03 | 9.45E-06 | 21.78 |
|  | rs2900503 | G | T | -0.17 | 0.03 | 1.55E-07 | 32.11 |
|  | rs2998141 | T | C | -0.14 | 0.03 | 4.42E-06 | 21.78 |
|  | rs4270579 | G | A | -0.12 | 0.03 | 5.46E-06 | 16 |
|  | rs4717843 | G | T | -0.12 | 0.03 | 4.72E-06 | 16 |
|  | rs7712231 | A | G | 0.16 | 0.04 | 7.97E-06 | 16 |
|  | rs80309088 | G | A | 0.17 | 0.04 | 4.56E-06 | 18.06 |
|  | rs9887954 | G | A | -0.11 | 0.02 | 4.81E-06 | 30.25 |
| RuminococcaceaeUCG010 | rs10414815 | T | C | 0.10 | 0.02 | 4.24E-06 | 25 |
|  | rs11192447 | A | G | 0.13 | 0.02 | 4.69E-07 | 42.25 |
|  | rs12346653 | C | T | 0.07 | 0.01 | 2.70E-06 | 49 |
|  | rs17730011 | G | A | -0.07 | 0.02 | 7.85E-06 | 12.25 |
|  | rs2833528 | C | T | -0.06 | 0.01 | 9.92E-06 | 36 |
|  | rs336138 | G | T | 0.08 | 0.02 | 7.48E-06 | 16 |
|  | rs4576377 | A | C | -0.06 | 0.01 | 7.63E-06 | 36 |
|  | rs72894957 | G | A | 0.22 | 0.05 | 5.68E-06 | 19.36 |
|  | rs74315802 | G | T | 0.09 | 0.02 | 3.19E-06 | 20.25 |
|  | rs9981767 | A | C | 0.07 | 0.01 | 9.96E-07 | 49 |
| RuminococcaceaeUCG003 | rs10490280 | C | T | -0.07 | 0.01 | 4.16E-06 | 49 |
|  | rs11243416 | T | C | -0.09 | 0.02 | 1.67E-06 | 20.25 |
|  | rs11613919 | G | T | 0.07 | 0.02 | 1.63E-06 | 12.25 |
|  | rs16959793 | A | C | -0.06 | 0.01 | 2.22E-06 | 36 |
|  | rs2523124 | T | C | -0.05 | 0.01 | 5.78E-06 | 25 |
|  | rs3013089 | G | A | -0.06 | 0.01 | 4.38E-06 | 36 |
|  | rs4452755 | A | C | -0.06 | 0.01 | 3.29E-06 | 36 |
|  | rs4532474 | G | A | 0.08 | 0.02 | 4.82E-06 | 16 |
|  | rs646327 | G | A | 0.06 | 0.01 | 7.83E-07 | 36 |
|  | rs6759615 | A | G | 0.10 | 0.02 | 7.86E-07 | 25 |
|  | rs73341549 | T | C | -0.17 | 0.03 | 1.51E-07 | 32.11 |
|  | rs78720113 | A | G | -0.12 | 0.02 | 7.59E-06 | 36 |
| Victivallis | rs11899949 | G | A | 0.13 | 0.03 | 2.77E-06 | 18.78 |
|  | rs12512543 | A | C | -0.18 | 0.04 | 2.54E-06 | 20.25 |
|  | rs173120 | T | C | 0.13 | 0.03 | 7.65E-06 | 18.78 |
|  | rs1882775 | A | G | -0.14 | 0.03 | 8.73E-06 | 21.78 |
|  | rs2546432 | T | C | -0.11 | 0.02 | 9.93E-06 | 30.25 |
|  | rs342302 | A | G | -0.15 | 0.04 | 8.16E-06 | 14.06 |
|  | rs4764863 | G | A | 0.12 | 0.02 | 8.22E-07 | 36 |
|  | rs4895919 | T | C | -0.12 | 0.02 | 2.75E-06 | 36 |
|  | rs56349194 | A | G | -0.16 | 0.03 | 6.26E-07 | 28.44 |
|  | rs911666 | T | C | -0.12 | 0.03 | 7.65E-06 | 16 |
| Methanobacteriales | rs10202904 | T | G | -0.12 | 0.02 | 3.01E-07 | 36 |
|  | rs10424197 | G | A | -0.11 | 0.02 | 9.28E-06 | 30.25 |
|  | rs4257531 | G | A | 0.16 | 0.04 | 7.44E-06 | 16 |
|  | rs62241835 | G | T | -0.20 | 0.04 | 1.63E-06 | 25 |
|  | rs6508769 | T | C | 0.15 | 0.03 | 8.23E-06 | 25 |
|  | rs73068003 | G | T | -0.16 | 0.04 | 8.45E-06 | 16 |
|  | rs73457410 | A | G | 0.22 | 0.04 | 1.41E-06 | 30.25 |
|  | rs75208022 | C | T | -0.23 | 0.05 | 5.92E-06 | 21.16 |
|  | rs894996 | C | A | 0.22 | 0.04 | 1.88E-06 | 30.25 |
| Euryarchaeota | rs10202904 | T | G | -0.12 | 0.02 | 6.19E-07 | 36 |
|  | rs11022995 | G | A | -0.10 | 0.02 | 7.73E-06 | 25 |
|  | rs34928225 | T | C | 0.20 | 0.04 | 4.33E-06 | 25 |
|  | rs45498998 | G | A | -0.13 | 0.03 | 5.32E-06 | 18.78 |
|  | rs6064552 | T | C | -0.12 | 0.03 | 9.34E-06 | 16 |
|  | rs6508769 | T | C | 0.15 | 0.03 | 8.12E-06 | 25 |
|  | rs7015093 | G | A | -0.12 | 0.03 | 7.20E-06 | 16 |
|  | rs76029318 | T | C | 0.21 | 0.04 | 1.05E-06 | 27.56 |
|  | rs7635189 | G | A | 0.12 | 0.03 | 4.64E-06 | 16 |
|  | rs77658038 | A | C | -0.16 | 0.03 | 4.75E-06 | 28.44 |
|  | rs894996 | C | A | 0.20 | 0.04 | 5.12E-06 | 25 |
| Proteobacteria | rs10750258 | A | C | -0.05 | 0.01 | 8.72E-06 | 25 |
|  | rs11126162 | T | C | -0.08 | 0.02 | 9.26E-06 | 16 |
|  | rs11715072 | G | A | -0.05 | 0.01 | 6.90E-06 | 25 |
|  | rs12150865 | C | T | 0.05 | 0.01 | 1.54E-06 | 25 |
|  | rs12467198 | C | T | 0.05 | 0.01 | 6.31E-06 | 25 |
|  | rs2347697 | G | T | 0.05 | 0.01 | 4.27E-06 | 25 |
|  | rs2532663 | G | A | -0.13 | 0.03 | 7.47E-07 | 18.78 |
|  | rs3890996 | T | G | -0.05 | 0.01 | 6.95E-06 | 25 |
|  | rs4340090 | C | T | -0.07 | 0.02 | 9.99E-06 | 12.25 |
|  | rs6707783 | C | T | 0.08 | 0.02 | 8.09E-06 | 16 |
|  | rs72771021 | C | T | 0.14 | 0.03 | 7.18E-06 | 21.78 |
|  | rs922773 | C | T | -0.08 | 0.02 | 3.68E-07 | 16 |

| **Exposure** | **Method** | **nsnp** | **Beta** | **SE** | **P value**  **(Global Test P value)** | **Egger intercept** | **P for pleiotropy** | **P for heterogeneity** |
| --- | --- | --- | --- | --- | --- | --- | --- | --- |
| Vitamin A | IVW | 2 | - | - | - |  |  |  |
| Vitamin B_12_ | IVW | 5 | -0.18 | 0.15 | 0.22 |  |  | 0.46 |
|  | MR Egger | 5 | -0.18 | 0.74 | 0.82 | 0.0003 | 0.996 |  |
|  | MR-PRESSO | 5 | -0.33 | 0.14 | 0.26 |  |  |  |
|  | Simple mode | 5 | -0.03 | 0.26 | 0.92 |  |  |  |
|  | Weighted median | 5 | -0.13 | 0.19 | 0.51 |  |  |  |
|  | Weighted mode | 5 | -0.04 | 0.26 | 0.89 |  |  |  |
| Vitamin C | IVW | 9 | 0.05 | 0.15 | 0.72 |  |  | 0.43 |
|  | MR Egger | 9 | -0.12 | 0.25 | 0.64 | 0.01 | 0.41 |  |
|  | MR-PRESSO | 9 | 0.002 | 0.15 | 0.41 |  |  |  |
|  | Simple mode | 9 | -0.18 | 0.33 | 0.60 |  |  |  |
|  | Weighted median | 9 | -0.07 | 0.18 | 0.71 |  |  |  |
|  | Weighted mode | 9 | -0.14 | 0.21 | 0.52 |  |  |  |
| Vitamin D | **IVW** | **8** | **-0.41** | **0.15** | **0.008** |  |  | **0.40** |
|  | MR Egger | 8 | -0.45 | 0.22 | 0.08 | 0.005 | 0.76 |  |
|  | MR-PRESSO | 8 | 0.46 | 0.19 | 0.21 |  |  |  |
|  | Simple mode | 8 | -0.55 | 0.30 | 0.11 |  |  |  |
|  | **Weighted median** | **8** | **-0.44** | **0.18** | **0.01** |  |  |  |
|  | **Weighted mode** | **8** | **-0.40** | **0.17** | **0.05** |  |  |  |
| Vitamin E | IVW | 1 | - | - | - |  |  |  |

EA, effective alleles；OA, other alleles

**Table S3** The results of Mendelian randomization and sensitivity analysis on the association between different circulating vitamin levels and cholecystitis.

IVW, Inverse variance weighting

**Table S4** The results of Mendelian randomization and sensitivity analysis on the association between gut microbiota and cholecystitis.

| Gut microbiota--Cholecystitis | | | | | | | | |
| --- | --- | --- | --- | --- | --- | --- | --- | --- |
| **Exposure** | **Method** | **nsnp** | **Beta** | **SE** | **P value**  **(Global Test P value)** | **Egger intercept** | **P for pleiotropy** | **P for heterogeneity** |
| Clostridia | IVW | 15 | -0.24 | 0.12 | 0.04 |  |  | 0.82 |
|  | MR Egger | 15 | -0.86 | 0.49 | 0.10 | 0.04 | 0.21 |  |
|  | MR-PRESSO | 15 | -0.24 | 0.10 | 0.81 |  |  |  |
|  | cML-MA | 15 | -0.25 | 0.12 | 0.04 |  |  |  |
|  | Weighted median | 15 | -0.23 | 0.17 | 0.17 |  |  |  |
| Methanobacteria | IVW | 11 | -0.15 | 0.07 | 0.02 |  |  | 0.82 |
|  | MR Egger | 11 | -0.34 | 0.28 | 0.28 | 0.04 | 0.21 |  |
|  | MR-PRESSO | 11 | -0.15 | 0.07 | 0.31 |  |  |  |
|  | cML-MA | 11 | -0.16 | 0.07 | 0.02 |  |  |  |
|  | Weighted median | 11 | -0.14 | 0.10 | 0.16 |  |  |  |
| FamilyXIII | IVW | 9 | -0.33 | 0.15 | 0.04 |  |  | 0.37 |
|  | MR Egger | 9 | 0.27 | 0.68 | 0.71 | -0.04 | 0.40 |  |
|  | MR-PRESSO | 9 | -0.33 | 0.16 | 0.39 |  |  |  |
|  | cML-MA | 9 | -0.34 | 0.16 | 0.04 |  |  |  |
|  | Weighted median | 9 | -0.37 | 0.21 | 0.08 |  |  |  |
| Methanobacteriaceae | IVW | 11 | -0.15 | 0.07 | 0.02 |  |  | 0.28 |
|  | MR Egger | 11 | -0.33 | 0.28 | 0.28 | 0.03 | 0.53 |  |
|  | MR-PRESSO | 11 | -0.15 | 0.07 | 0.33 |  |  |  |
|  | cML-MA | 11 | -0.16 | 0.07 | 0.02 |  |  |  |
|  | Weighted median | 11 | -0.14 | 0.10 | 0.15 |  |  |  |
| Coprococcus3 | IVW | 8 | 0.40 | 0.16 | 0.02 |  |  | 0.54 |
|  | MR Egger | 8 | 0.86 | 0.91 | 0.38 | -0.03 | 0.62 |  |
|  | MR-PRESSO | 8 | 0.40 | 0.15 | 0.54 |  |  |  |
|  | cML-MA | 8 | 0.41 | 0.17 | 0.02 |  |  |  |
|  | Weighted median | 8 | 0.26 | 0.21 | 0.22 |  |  |  |
| Odoribacter | IVW | 8 | -0.42 | 0.15 | 0.005 |  |  | 0.80 |
|  | MR Egger | 8 | 0.06 | 0.47 | 0.90 | -0.04 | 0.32 |  |
|  | MR-PRESSO | 8 | -0.42 | 0.11 | 0.79 |  |  |  |
|  | cML-MA | 8 | -0.43 | 0.16 | 0.005 |  |  |  |
|  | Weighted median | 8 | -0.39 | 0.20 | 0.05 |  |  |  |
| RikenellaceaeRC9gutgroup | IVW | 12 | -0.13 | 0.06 | 0.03 |  |  | 0.63 |
|  | MR Egger | 12 | 0.41 | 0.38 | 0.31 | -0.08 | 0.18 |  |
|  | MR-PRESSO | 12 | -0.13 | 0.05 | 0.64 |  |  |  |
|  | cML-MA | 12 | -0.14 | 0.06 | 0.03 |  |  |  |
|  | Weighted median | 12 | -0.15 | 0.08 | 0.07 |  |  |  |
| RuminococcaceaeUCG010 | IVW | 8 | -0.26 | 0.13 | 0.05 |  |  | 0.70 |
|  | MR Egger | 8 | -0.30 | 0.41 | 0.49 | 0.05 | 0.08 |  |
|  | MR-PRESSO | 8 | -0.26 | 0.06 | 0.99 |  |  |  |
|  | cML-MA | 8 | -0.26 | 0.14 | 0.05 |  |  |  |
|  | Weighted median | 8 | -0.31 | 0.17 | 0.06 |  |  |  |
| **RuminococcaceaeUCG003** | **IVW** | **14** | **0.22** | **0.11** | **0.04** |  |  | **0.98** |
|  | **MR Egger** | **14** | **-0.44** | **0.36** | **0.24** | **0.003** | **0.93** |  |
|  | **MR-PRESSO** | **14** | **0.22** | **0.09** | **0.68** |  |  |  |
|  | **cML-MA** | **14** | **0.23** | **0.11** | **0.04** |  |  |  |
|  | **Weighted median** | **14** | **0.23** | **0.14** | **0.09** |  |  |  |
| Victivallis | IVW | 12 | 0.12 | 0.06 | 0.04 |  |  | 0.44 |
|  | MR Egger | 12 | -0.14 | 0.41 | 0.75 | 0.03 | 0.54 |  |
|  | MR-PRESSO | 12 | 0.12 | 0.06 | 0.50 |  |  |  |
|  | cML-MA | 12 | 0.13 | 0.06 | 0.04 |  |  |  |
|  | Weighted median | 12 | 0.14 | 0.08 | 0.09 |  |  |  |
| Methanobacteriales | IVW | 11 | -0.15 | 0.07 | 0.02 |  |  | 0.28 |
|  | MR Egger | 11 | -0.33 | 0.28 | 0.28 | 0.03 | 0.53 |  |
|  | MR-PRESSO | 11 | -0.15 | 0.07 | 0.32 |  |  |  |
|  | cML-MA | 11 | -0.16 | 0.07 | 0.02 |  |  |  |
|  | Weighted median | 11 | -0.14 | 0.09 | 0.14 |  |  |  |
| Euryarchaeota | IVW | 11 | -0.14 | 0.07 | 0.03 |  |  | 0.08 |
|  | MR Egger | 11 | -0.28 | 0.39 | 0.50 | 0.02 | 0.72 |  |
|  | MR-PRESSO | 11 | -0.14 | 0.08 | 0.10 |  |  |  |
|  | cML-MA | 11 | -0.15 | 0.07 | 0.03 |  |  |  |
|  | Weighted median | 11 | -0.10 | 0.10 | 0.30 |  |  |  |
| Proteobacteria | IVW | 14 | -0.29 | 0.12 | 0.01 |  |  | 0.48 |
|  | MR Egger | 14 | -1.11 | 0.35 | 0.007 | 0.06 | 0.03 |  |
|  | MR-PRESSO | 14 | -0.29 | 0.12 | 0.46 |  |  |  |
|  | cML-MA | 14 | -0.31 | 0.12 | 0.01 |  |  |  |
|  | Weighted median | 14 | -0.18 | 0.17 | 0.28 |  |  |  |

IVW, Inverse variance weighting

**Table S5** The results of Mendelian randomization and sensitivity analysis on the association between gut microbiota and circulating vitamin D levels.

| Gut microbiota--Vitamin D | | | | | | | | |
| --- | --- | --- | --- | --- | --- | --- | --- | --- |
| **Exposure** | **Method** | **nsnp** | **Beta** | **SE** | **P value**  **(Global Test P value)** | **Egger intercept** | **P for pleiotropy** | **P for heterogeneity** |
| Coriobacteriia | IVW | 14 | -0.04 | 0.01 | 0.004 |  |  | 0.19 |
|  | MR Egger | 14 | -0.10 | 0.06 | 0.13 | 0.004 | 0.33 |  |
|  | Weighted median | 14 | -0.02 | 0.02 | 0.37 |  |  |  |
|  | cML-MA | 14 | -0.04 | 0.02 | 0.007 |  |  |  |
|  | MR-PRESSO | 14 | -0.04 | 0.02 | 0.19 |  |  |  |
| Coriobacteriaceae | IVW | 14 | -0.04 | 0.01 | 0.004 |  |  | 0.19 |
|  | MR Egger | 14 | -0.10 | 0.06 | 0.13 | 0.004 | 0.33 |  |
|  | Weighted median | 14 | -0.02 | 0.02 | 0.34 |  |  |  |
|  | cML-MA | 14 | -0.04 | 0.02 | 0.007 |  |  |  |
|  | MR-PRESSO | 14 | -0.04 | 0.02 | 0.19 |  |  |  |
| Lactobacillaceae | IVW | 9 | -0.03 | 0.01 | 0.04 |  |  | 0.19 |
|  | MR Egger | 9 | -0.03 | 0.05 | 0.57 | 4.38E-5 | 0.99 |  |
|  | Weighted median | 9 | -0.04 | 0.02 | 0.02 |  |  |  |
|  | cML-MA | 9 | -0.03 | 0.01 | 0.04 |  |  |  |
|  | MR-PRESSO | 9 | -0.03 | 0.02 | 0.32 |  |  |  |
| Prevotellaceae | IVW | 16 | -0.02 | 0.01 | 0.04 |  |  | 0.19 |
|  | MR Egger | 16 | -0.02 | 0.04 | 0.69 | -0.0005 | 0.87 |  |
|  | Weighted median | 16 | -0.03 | 0.02 | 0.10 |  |  |  |
|  | cML-MA | 16 | -0.03 | 0.01 | 0.04 |  |  |  |
|  | MR-PRESSO | 16 | -0.02 | 0.01 | 0.24 |  |  |  |
| Anaerotruncus | IVW | 13 | -0.05 | 0.02 | 0.003 |  |  | 0.97 |
|  | MR Egger | 13 | -0.08 | 0.07 | 0.26 | 0.003 | 0.59 |  |
|  | Weighted median | 13 | -0.04 | 0.02 | 0.06 |  |  |  |
|  | cML-MA | 13 | -0.05 | 0.02 | 0.003 |  |  |  |
|  | MR-PRESSO | 13 | -0.05 | 0.009 | 0.97 |  |  |  |
| Escherichia | IVW | 10 | 0.03 | 0.02 | 0.04 |  |  | 0.61 |
|  | MR Egger | 10 | 0.05 | 0.07 | 0.53 | -0.0009 | 0.86 |  |
|  | Weighted median | 10 | 0.04 | 0.02 | 0.06 |  |  |  |
|  | cML-MA | 10 | 0.03 | 0.02 | 0.04 |  |  |  |
|  | MR-PRESSO | 10 | 0.03 | 0.01 | 0.74 |  |  |  |
| LachnospiraceaeND3007group | IVW | 3 | 0.08 | 0.03 | 0.02 |  |  | 0.07 |
|  | MR Egger | 3 | 0.008 | 2.54 | 0.998 | 0.004 | 0.98 |  |
|  | Weighted median | 3 | 0.06 | 0.05 | 0.25 |  |  |  |
|  | cML-MA | 3 | 0.08 | 0.04 | 0.04 |  |  |  |
|  | MR-PRESSO | 3 | -0.03 | 0.01 | 0.40 |  |  |  |
| Lactobacillus | IVW | 10 | -0.03 | 0.01 | 0.02 |  |  | 0.32 |
|  | MR Egger | 10 | -0.05 | 0.05 | 0.30 | 0.002 | 0.62 |  |
|  | Weighted median | 10 | -0.04 | 0.02 | 0.03 |  |  |  |
|  | cML-MA | 10 | -0.03 | 0.01 | 0.02 |  |  |  |
|  | MR-PRESSO | 10 | -0.03 | 0.01 | 0.44 |  |  |  |
| Oscillibacter | IVW | 14 | -0.03 | 0.01 | 0.03 |  |  | 0.46 |
|  | MR Egger | 14 | -0.04 | 0.04 | 0.43 | 0.001 | 0.82 |  |
|  | Weighted median | 14 | -0.02 | 0.02 | 0.22 |  |  |  |
|  | cML-MA | 14 | -0.03 | 0.01 | 0.02 |  |  |  |
|  | MR-PRESSO | 14 | -0.03 | 0.01 | 0.43 |  |  |  |
| Parasutterella | IVW | 15 | -0.03 | 0.01 | 0.01 |  |  | 0.60 |
|  | MR Egger | 15 | 0.09 | 0.07 | 0.23 | -0.008 | 0.13 |  |
|  | Weighted median | 15 | -0.02 | 0.01 | 0.08 |  |  |  |
|  | cML-MA | 15 | -0.03 | 0.01 | 0.01 |  |  |  |
|  | MR-PRESSO | 15 | -0.03 | 0.01 | 0.69 |  |  |  |
| **RuminococcaceaeUCG003** | **IVW** | **12** | **-0.03** | **0.01** | **0.04** |  |  | **0.31** |
|  | **MR Egger** | **12** | **-0.20** | **0.08** | **0.03** | **0.01** | **0.06** |  |
|  | **Weighted median** | **12** | **-0.05** | **0.02** | **0.02** |  |  |  |
|  | **cML-MA** | **12** | **-0.03** | **0.02** | **0.04** |  |  |  |
|  | **MR-PRESSO** | **12** | **-0.03** | **0.02** | **0.31** |  |  |  |
| RuminococcaceaeUCG013 | IVW | 12 | -0.04 | 0.02 | 0.02 |  |  | 0.40 |
|  | MR Egger | 12 | -0.06 | 0.07 | 0.39 | 0.002 | 0.75 |  |
|  | Weighted median | 12 | -0.04 | 0.03 | 0.13 |  |  |  |
|  | cML-MA | 12 | -0.04 | 0.02 | 0.02 |  |  |  |
|  | MR-PRESSO | 12 | -0.04 | 0.02 | 0.45 |  |  |  |
| Streptococcus | IVW | 15 | -0.03 | 0.02 | 0.05 |  |  | 0.58 |
|  | MR Egger | 15 | -0.07 | 0.08 | 0.38 | 0.003 | 0.64 |  |
|  | Weighted median | 15 | -0.03 | 0.02 | 0.23 |  |  |  |
|  | cML-MA | 15 | -0.04 | 0.02 | 0.05 |  |  |  |
|  | MR-PRESSO | 15 | -0.03 | 0.02 | 0.64 |  |  |  |
| unknowngenus | IVW | 16 | 0.03 | 0.02 | 0.03 |  |  | 0.80 |
|  | MR Egger | 16 | -0.04 | 0.07 | 0.62 | 0.005 | 0.34 |  |
|  | Weighted median | 16 | 0.03 | 0.02 | 0.09 |  |  |  |
|  | cML-MA | 16 | 0.03 | 0.02 | 0.03 |  |  |  |
|  | MR-PRESSO | 16 | 0.03 | 0.01 | 0.78 |  |  |  |
| Coriobacteriales | IVW | 14 | -0.04 | 0.01 | 0.004 |  |  | 0.19 |
|  | MR Egger | 14 | -0.10 | 0.06 | 0.13 | 0.004 | 0.33 |  |
|  | Weighted median | 14 | -0.02 | 0.02 | 0.35 |  |  |  |
|  | cML-MA | 14 | -0.04 | 0.02 | 0.007 |  |  |  |
|  | MR-PRESSO | 14 | -0.04 | 0.02 | 0.20 |  |  |  |

IVW, Inverse variance weighting

**Table S6** Instrumental variable information used for reverse Mendelian randomization on cholecystitis.

| Exposure | SNP | OA | EA | beta | se | pval |
| --- | --- | --- | --- | --- | --- | --- |
| Cholecystitis | rs1417965 | A | G | -0.12 | 0.03 | 4.88E-06 |
|  | rs11800918 | C | T | -0.29 | 0.06 | 9.22E-06 |
|  | rs78621185 | T | C | 0.29 | 0.06 | 8.70E-06 |
|  | rs11887534 | G | C | 0.28 | 0.04 | 2.06E-12 |
|  | rs10204966 | C | T | -0.12 | 0.03 | 9.68E-06 |
|  | rs62190055 | C | T | -0.3 | 0.07 | 3.36E-06 |
|  | rs12623271 | C | G | 0.14 | 0.02 | 1.56E-09 |
|  | rs148388002 | G | T | 0.41 | 0.09 | 7.01E-06 |
|  | rs139778630 | C | T | -0.36 | 0.08 | 1.58E-06 |
|  | rs137977673 | A | G | 0.26 | 0.06 | 7.35E-06 |
|  | rs143332484 | C | T | -0.77 | 0.17 | 4.48E-06 |
|  | rs73055219 | T | G | -0.14 | 0.03 | 5.47E-06 |
|  | rs144484680 | G | A | -0.25 | 0.05 | 1.39E-06 |
|  | rs2937369 | C | G | -0.11 | 0.02 | 8.43E-07 |
|  | rs628094 | G | A | 0.12 | 0.03 | 2.32E-06 |
|  | rs2384533 | T | C | 0.11 | 0.02 | 1.46E-06 |
|  | rs61858780 | C | A | 0.34 | 0.07 | 1.30E-06 |
|  | rs67033524 | A | G | -0.16 | 0.03 | 3.62E-06 |
|  | rs2367073 | A | G | -0.14 | 0.03 | 3.26E-06 |
|  | rs146423587 | G | A | 0.32 | 0.07 | 4.00E-06 |
|  | rs12860390 | T | C | -0.33 | 0.07 | 6.29E-06 |
|  | rs12590849 | A | G | -0.11 | 0.02 | 6.92E-06 |
|  | rs4887243 | C | T | 0.13 | 0.03 | 3.71E-06 |
|  | rs4620933 | A | C | 0.12 | 0.03 | 2.11E-06 |
|  | rs141295428 | G | A | 0.34 | 0.08 | 3.33E-06 |
|  | rs117434865 | C | T | 0.93 | 0.21 | 9.96E-06 |
|  | rs12962445 | C | T | 0.16 | 0.03 | 3.74E-06 |
|  | rs10422977 | C | T | 0.14 | 0.03 | 7.30E-07 |
|  | rs2207417 | C | T | -0.54 | 0.12 | 6.49E-06 |
|  | rs739067 | G | A | 0.35 | 0.08 | 4.74E-06 |

EA, effective alleles；OA, other alleles

## 2 Supplementary Figures


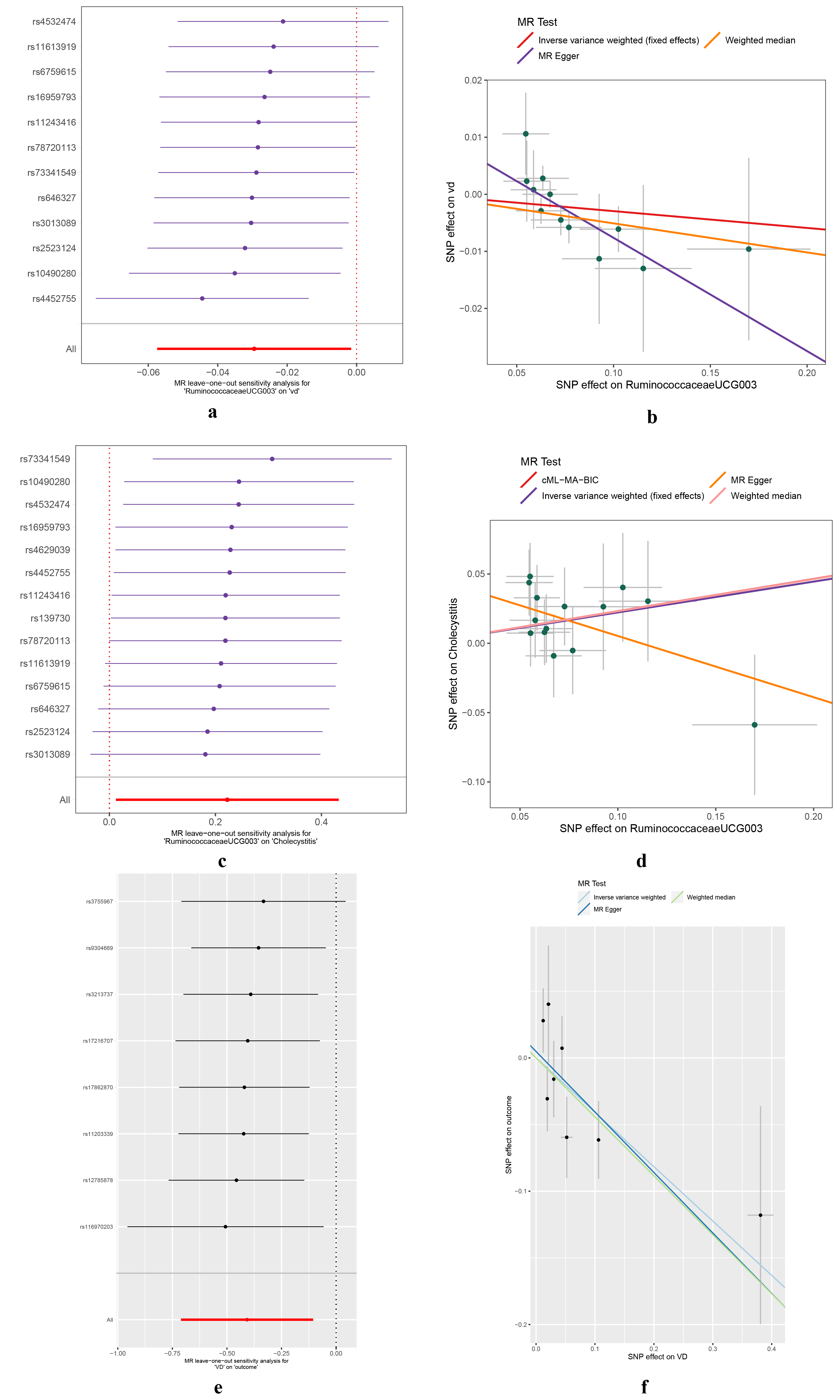


a. The Mendelian randomization results of the gut microbiota genus RuminococcaceaeUCG003 with circulating vitamin D levels; b. The leave-one-out results of the gut microbiota genus RuminococcaceaeUCG003 with circulating vitamin D levels; c. The Mendelian randomization results of the gut microbiota genus RuminococcaceaeUCG003 with cholecystitis; d. The leave-one-out results of the gut microbiota genus RuminococcaceaeUCG003 with cholecystitis; e. The Mendelian randomization results of circulating vitamin D levels with cholecystitis; f. The leave-one-out results of circulating vitamin D levels with cholecystitis.

**Figure S1** Visualization of the main results of the two-step Mendelian randomization in this study includes scatter plots and leave-one-out analysis.
